# Sulfated Flavonoids from *Phyllospadix* (Zosteraceae) Taxa from Baja California, Mexico

**DOI:** 10.1021/acsomega.4c10402

**Published:** 2025-02-07

**Authors:** Diego Rodríguez-Hernández, Jose Miguel Sandoval-Gil, Kjersti Hasle Enerstvedt, Susana Villa Gonzalez, Alejandra Ferreira-Arrieta, Alexandros G. Asimakopoulos, Monica Jordheim

**Affiliations:** 1 Department of Chemistry, University of Bergen, Allegaten 41, Bergen NO-5007, Norway; 2 Instituto de Investigaciones Oceanólogicas, Universidad Autónoma de Baja California, Ensenada, Baja California 22830, Mexico; 3 Department of Chemistry, Norwegian University of Science and Technology (NTNU), Trondheim 7491, Norway

## Abstract

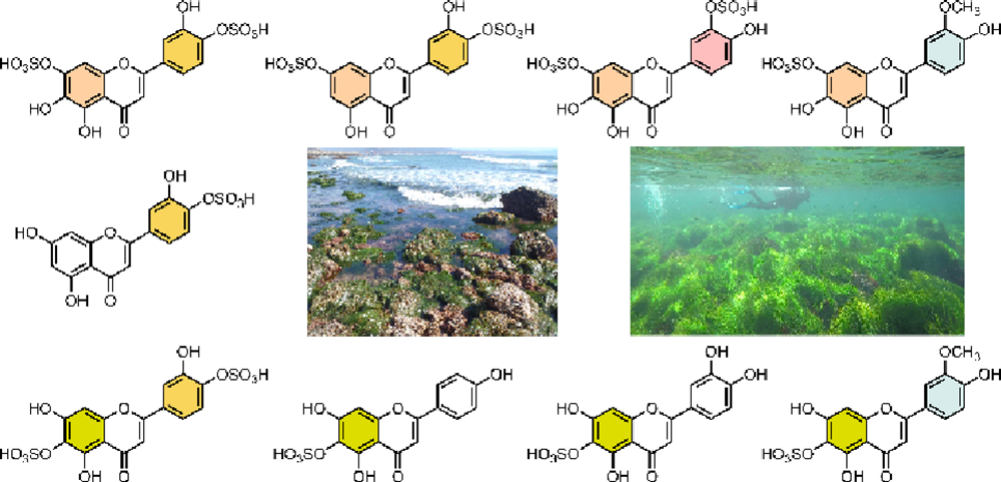

Sulfated flavonoids, a class of polyphenols integral to plant secondary metabolism and chemical defense, exhibit notable pharmacological potential. Seagrasses, marine angiosperms with critical ecological and socioeconomic roles, often accumulate these compounds in high concentrations. However, their complex chemical profiles—including closely related sulfated flavonoids—are challenging to characterize due to potential degradation during extraction. This study provides the first comprehensive analysis of sulfated flavonoids in *Phyllospadix scouleri* alongside a comparative analysis of *P. torreyi*. The *Phyllospadix* genus, known for forming productive intertidal meadows on rocky Pacific coastlines of North America, serves as a valuable model for understanding flavonoid diversity and adaptation in marine environments. From *P. scouleri* foliar tissues, we isolated and identified 1 phenolic acid and 15 sulfated flavonoids (HPLC-DAD, NMR, LC-MS), including previously undescribed 6-hydroxyflavonoid disulfates and monosulfates, and flavonoids not earlier reported for the genus. Lower amounts of sulfated glycosides were also tentatively identified in both species for the first time. The flavonoid profiles showed clear species-specific patterns: *P. scouleri* primarily produced 6-hydroxyflavonoids (70%), while *P. torreyi* favored 5- and 6-methoxyflavonoids (60 and 70%). Samples collected from nearby locations in May 2024 from both species showed similar flavonoid concentrations (∼20 mg/g DW) and comparable ratios between total flavonoids and rosmarinic acid (∼6:1). *P. torreyi* exhibited more disulfated flavonoids (84.3%) than monosulfated types (11.9%), whereas *P. scouleri* had 25.2% disulfated and 66.5% monosulfated flavonoids. Given the proven link between phenolic compounds and the physiological acclimation of surfgrasses to emersion during intertidal periods, as well as to marine heatwaves, this study provides a robust baseline for further research into the basic chemical ecology of these compounds and their responses to climate change.

## Introduction

Sulfated flavonoids are bioactive compounds that play essential roles in terrestrial plants’ physiological and ecological processes, contributing to stress responses, defense mechanisms, and ecosystem interactions.^1−3^ Although less common, these compounds are also found in marine angiosperms, known as seagrasses, where they may serve protective roles against pathogens, mitigate environmental stress, and influence plant interactions within marine ecosystems.^4−8^ While sulfated flavonoids are crucial for seagrass survival, their functions vary among species, and their ecological contexts are still uncertain.

Within marine ecosystems, species of the *Phyllospadix* (surfgrasses) are notable for their ecological importance.^9,10^ The genus includes five species: *Phyllospadix japonicus* Makino and *P. iwatensis* Makino, native to Asia, and *P. serrulatus*, *P. scouleri* Hooker, and *P. torreyi* S. Watson, found along the Pacific coast of North America.^11^ Sulfated flavonoids have been found in all five Phyllospadix species.^12−14^ However, the results are based solely on their electrophoretic mobility on paper, and have not been isolated and characterized.^8^ For the Asian species, a mixture of eight flavonoids was isolated from *P. japonicus*, including 6-hydroxyluteolin, 5-methoxyluteolin, hispidulin (6-methoxyapigenin), jaceosidin (5-methoxychryseriol), 6-methoxydiosmetin, acacetin 5-methyl ether, and acacetin 6-methyl ether.^15^ Hispidulin, luteolin, and phyllospadine, a new flavonoidal alkaloid, were also found in *P. iwatensis*.^14^ Luteolin 7-sulfate and hispidulin 7-sulfate have recently been identified as additional constituents of *P. iwatensis*.^13^

In North America, *P. scouleri* and *P. torreyi*, also known as surfgrasses, are endemic to the northeastern Pacific, forming extensive meadows along rocky intertidal zones. These surfgrasses exhibit unique morphological adaptations that enable them to persist in high-energy coastal environments.^16,17^*P. torreyi* ranges from the rocky coast of Vancouver Island, Canada, to the Baja California peninsula in Mexico, while *P. scouleri* extends as far north as Sitka, Alaska.^18^ The productivity of surfgrass meadows is considerable,^10,19^ and this productivity plays an important role in supporting key ecological functions. These include carbon sequestration, urban wastewater filtration, and biodiversity maintenance.^20−22^ The wide latitudinal distribution and resilience of *P. scouleri* and *P. torreyi* to drastic shifts in light, temperature, and nutrient levels highlight their adaptability and importance in coastal ecosystems.^23,24^ Studies suggest that phenolic compounds in *P. torreyi* may be linked to stress tolerance, particularly during low tides when exposure to air is prolonged.^16^ In *P. scouleri*, changes in total phenolics have been linked to physiological stress as a result of the effects of marine heat waves.^37^ Recently, Grignon-Dubois et al.^12^ reported that *P. torreyi* collected in La Jolla (California, USA) contains 13 (isolated and characterized) sulfated flavonoids, including nepetin (6-methoxyluteolin) 7,4'-disulfate, 5-methoxyluteolin 7,3'-disulfate, 6-hydroxyluteolin-disulfate, luteolin 7,3'-disulfate, nepetin 7,3'-disulfate, 5-methoxyluteolin 7-sulfate, 6-hydroxyluteolin 7-sulfate, luteolin 7-sulfate, luteolin 3'-sulfate, nepetin 3'-sulfate, hispidulin 7-sulfate, and jaceosidin 7-sulfate. Their study noted a consistent phenolic profile over three years, with disulfated flavonoids comprising 70–90% of the total flavonoid content—a finding that emphasize these compounds’ prevalence and potential ecological roles in surfgrasses. Intriguingly, nonsulfated flavonoids were only observed in *P. torreyi* after hydrolysis of crude extracts, in contrast to findings in Asian *Phyllospadix* species.

This study expands on previous research by investigating sulfated flavonoids in North American *Phyllospadix*. We report the isolation and structural elucidation of sulfated flavonoids from the foliar tissues of *P. scoluleri* and *P. torreyi* plants collected in Baja California, Mexico. We also explore the potential ecological implications of these flavonoids, contributing to our understanding of the chemical adaptations surfgrasses employ to thrive in dynamic and often harsh marine environments.

## Results and Discussion

### Analysis of Extracts of *Phyllospadix* Species by HPLC-DAD and HR-LC/MS

**Figure 1:**
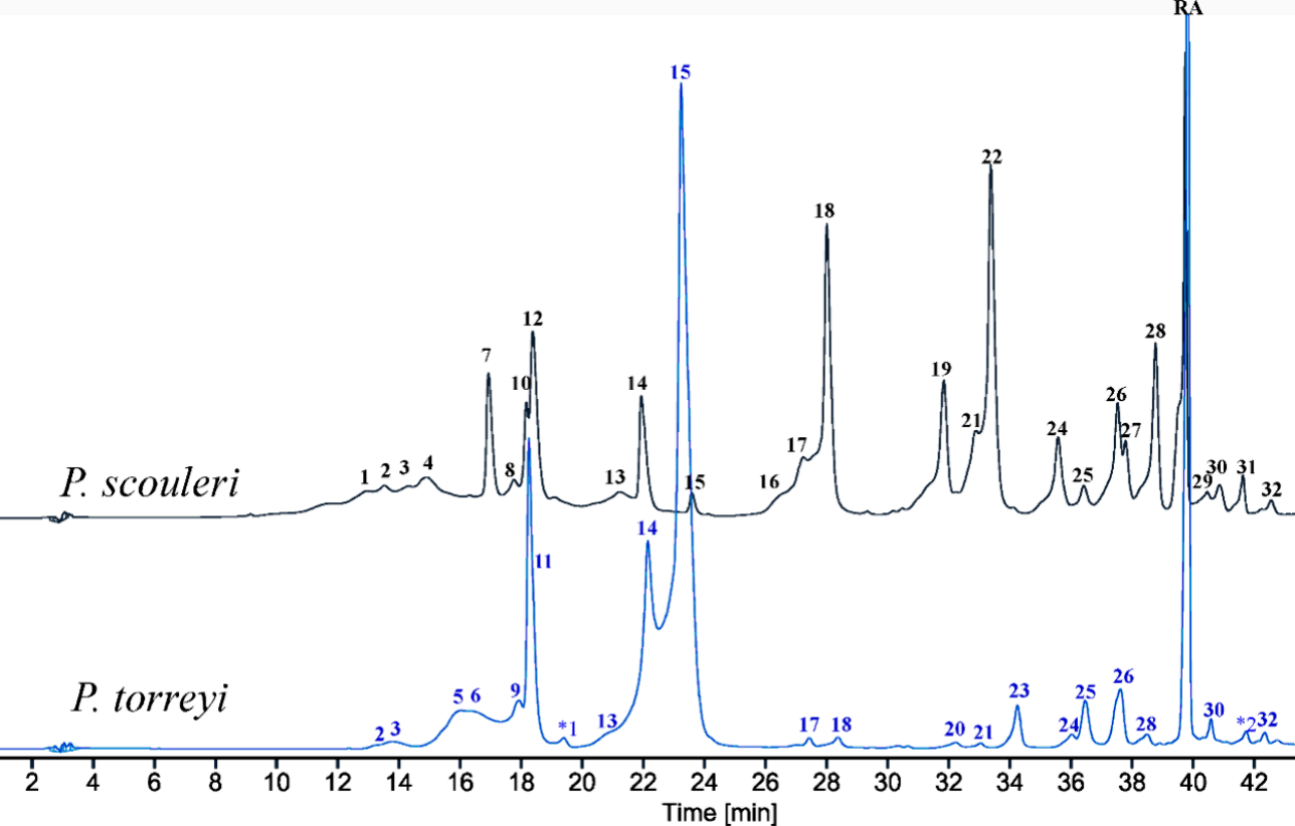
Representative HPLC profiles of crude extracts of *P. scouleri* and *P. torreyi* (recorded at 330 ± 20 nm). Assignment (^t^= tentative): **1**^t^: hispudilin sulfate-diglycoside; **2**^t^: 5-methoxyapigenin sulfate-diglycoside; **3**^t^: 5-methoxyapigenin sulfate-monoglycoside; **4**^t^: hispudilin sulfate-glycoside; **5**^t^: 5-methoxyluteolin sulfate-diglycoside; **6**: 5-methoxyluteolin sulfate-glycoside; **7**: 6-hydroxyluteolin 6,3'-disulfate; **8**^t^: 6-hydroxyluteolin disulfate; **9**^t^: 5-methoxyluteolin disulfate; **10**: 6-hydroxyluteolin 7,4'-disulfate; **11**: 5-methoxyluteolin 7,3'-disulfate; **12**: 6-hydroxyluteolin 7,3'-disulfate; **13**: luteolin 7,4'-disulfate; **14**: luteolin 7,3'-disulfate; **15**: nepetin 7,3'-disulfate; **16**: 6-hydroxyluteolin 6-sulfate; **17**: 6-hydroxyluteolin 7-sulfate; **18**: scutellarein 6-sulfate; **19**: nodifloretin 6-sulfate; **20**: nepetin 7-sulfate; **21**: luteolin sulfate; **22**: nodifloretin 7-sulfate; **23**^t^: nepetin sulfate; **24**: luteolin 4'-sulfate; **25**: luteolin 3'-sulfate; **26**: hispidulin 7-sulfate; **27**^**t**^: 6-hydroxyluteolin sulfate; **28**: jaceosidin 7-sulfate; **RA**: rosmarinic acid; **29**^t^: hispidulin; **30**^t^: 5-methoxyapigenin; **31**: 6-hydroxyluteolin; **32**: luteolin; (*^1^ = unknown flavonoid; *2 = unknown phenolic acid - deduced from UV–vis data).

Crude extracts of *Phyllospadix* species (*P. scouleri* and *P. torreyi*) were analyzed using HPLC-DAD to obtain their qualitative chromatographic profiles (Figure 1) along with HR-LR/MS in positive mode providing online UV spectra and mass spectral data for component identification (Table 1). Thirty-two (**1**-**32**) different flavonoids were observed of variable intensity detected at 330 ± 20 nm, in addition to rosmarinic acid (**RA**) (Figure 1).

**Table 1:** Qualitative and Quantitative Flavonoid and RA Content in *P. scouleri* and *P. torreyi*^a^

compound	HPLC, t_R_ (min)	UV_max_ (nm)	[M + H]^+^ (*m*/*z*)^R^	[F + H]^+^ (*m*/*z*)	*P. scouleri* (2024)^Q^	*P. torreyi* (2024)^Q^
**1**	12.96	333, 280	705	543, 381, 301	0.54 ± 0.33	
**2**	13.53	330, 267	689	527, 365, 285	*t*	*t*
**3**	14.32	330, 266	527	365, 285	*t*	*t*
**4**	14.9	333, 280	543	381, 301	0.67 ± 0.12	
**5**	15.30	332, 264	705	543, 381, 301		0.6 ± 0.08
**6**	16.04	332, 264	543	381, 301		c
**7**	16.94	328, 272	463.964	383, 303	0.83 ± 0.05	
**8**	17.77	330, 278	463	383, 303	0.27 ± 0.03	
**9**	17.94	332, 266	461	383, 301		0.53 ± 0.06
**10**	18.17	330, 280	463.9642	383, 303	0.93 ± 0.06	
**11**	1825	332, 266	461	381, 301		1.85 ± 0.07
**12**	18.38	332, 282	463.9640	383, 303	1.31 ± 0.04	
**13**	21.23	335, 282	447.9696	367, 287	0.35 ± 0.06	*t*
**14**	21.94	320, 270	447.9696	367, 287	1.07 ± 0.02	4.66 ± 0.11
**15**	23.59	332, 274	477.9796	397, 317	0.28 ± 0.01	10.39 ± 0.18
**16**	27.24	338, 274	383.0081	303	0.36 ± 0.06	
**17**	28.01	346, 282	383.0081	303	0.47 ± 0.05	0.04 ± 0.01
**18**	28.25	338, 274	367.0135	287	0.97 ± 0.09	0.09 ± 0.01
**19**	31.83	334, 282	397.0230	317	1.57 ± 0.16	
**20**	32.23	344, 282	397	317		0.05 ± 0.01
**21**	32.88	346, 280	367	287	0.57 ± 0.17	*t*
**22**	33.37	344, 282	397.0230	317	5.00 ± 0.35	
**23**	34.25	344, 272	397	317		0.38 ± 0.07
**24**	35.57	332, 268	367.0135	287	0.79 ± 0.10	0.19 ± 0.04
**25**	36.42	336, 268	367	287		0.47 ± 0.06
**26**	37.65	336, 272	381.0291	301	0.71 ± 0.01	0.57 ± 0.06
**27**	37.78	348, 270			0.39 ± 0.17	
**28**	38.76	346, 272	411.0381	331	1.27 ± 0.05	0.08 ± 0.01
**RA**	39.73	330	361		3.01 ± 0.08	3.89 ± 0.04
**29**	40.46	340, 274	301		0.40 ± 0.04	
**30**	40.86	332, 266	285		0.55 ± 0.5	0.64 ± 0.04
**31**	41.62	344, 280	303		0.62 ± 0.01	
**32**	42.55	326, 282	287		0.11 ± 0.02	0.15 ± 0.01
**total Fl**					20.03 ± 0.23	20.69 ± 0.15
% Fl sulfates					91.7	96.2
% Fl disulfates					25.2	84.3
% Fl monosulf.					66.5	11.9
% 6-OH					70	
% 5-OMe/6-OMe						60/70

a ^R^HR-MS (four digits) and LR-MS (no digits); Q = mg luteolin or RA eq./g dry weight (DW); *t* = traces (<0.5 area), c = quantified together with **5** due to coelution.

The *P. scouleri* samples from February 2022 and May 2024 exhibited similar qualitative phenolic profiles (Figure S1). Some quantitative differences were observed between the two collections, particularly in the first 14 eluting peaks, which showed a higher occurrence in the specimen collected in winter (2022). However, the limited number of collections in this study is insufficient to conclude seasonal changes at the molecular level. Among the flavonoids identified, 25.2% were disulfated in *P. scouleri* and 84.3% in *P. torreyi*, while monosulfated compounds accounted for 66.5% in *P. scouleri* and 11.9% in *P. torreyi* (Table 1). In *P. torreyi*, the flavonoid pattern was predominantly composed of three disulfated flavonoids: nepetin 7,3'-disulfate (**15**) was the most abundant compound, making up 50% of the total, followed by luteolin 7,3'-disulfate (**14**) at 22% and 5-methoxyluteolin 7,3'-disulfate (**11**) at 9%, aligning with findings reported by Grignon-Dubois et al.^12^ Additionally, *P. torreyi* showed a higher tendency toward producing 5- and 6-methoxyflavonoids (60% and 70%, respectively). In contrast, *P. scouleri* predominantly synthesized 6-hydroxyflavonoids (70%) and exhibited a more diverse flavonoid profile, dominated by seven main compounds. These included three disulfated flavonoids: 6-hydroxyluteolin 7,3'-disulfate (**12**; 6.5%), luteolin 7,3'-disulfate (**14**; 5.3%), and 6-hydroxyluteolin 7,4'-disulfate (**10**; 4.6%). The four main monosulfated flavonoids were led by nodifloretin 7-sulfate (**22**), comprising 25% of the total, followed by nodifloretin 6-sulfate (**19**) at 7.8%, jaceosidin 7-sulfate (**28**) at 6.3%, and scutellarein 6-sulfate (**18**) at 4.8%. Both species showed similar total flavonoid to rosmarinic acid (**RA**) ratios (∼6:1), with 15% in *P. scouleri* and 19% in *P. torreyi* (Table 1*)*. In addition to the high levels of rosmarinic acid (**RA**), the HPLC profile (Figure 1) revealed only one unidentified late-eluting compound (*2) resembling a phenolic acid UV spectrum in *P. torreyi*. However, trace amounts of phenolic acids may be present in both species. Additionally, sulfated glycosides were tentatively identified for the first time in small amounts in *P. scouleri* (**1**-**4**) and *P. torreyi* (**2**, **3**, **5**, and **6**) using UV data and LR-LCMS.^25−27^ Isolating and identifying the substitution patterns proved challenging due to the low compound quantities and similar retention times. This issue was also encountered with compounds **8**, **9**, **21**, **23**, and the later-eluting aglycones (**29**-**32**) in the HPLC profiles (Figure 1, Table 1).

### Isolation and Structural Characterization of Sulfated Flavonoid from *P. scouleri* Extracts

**Figure 2:**
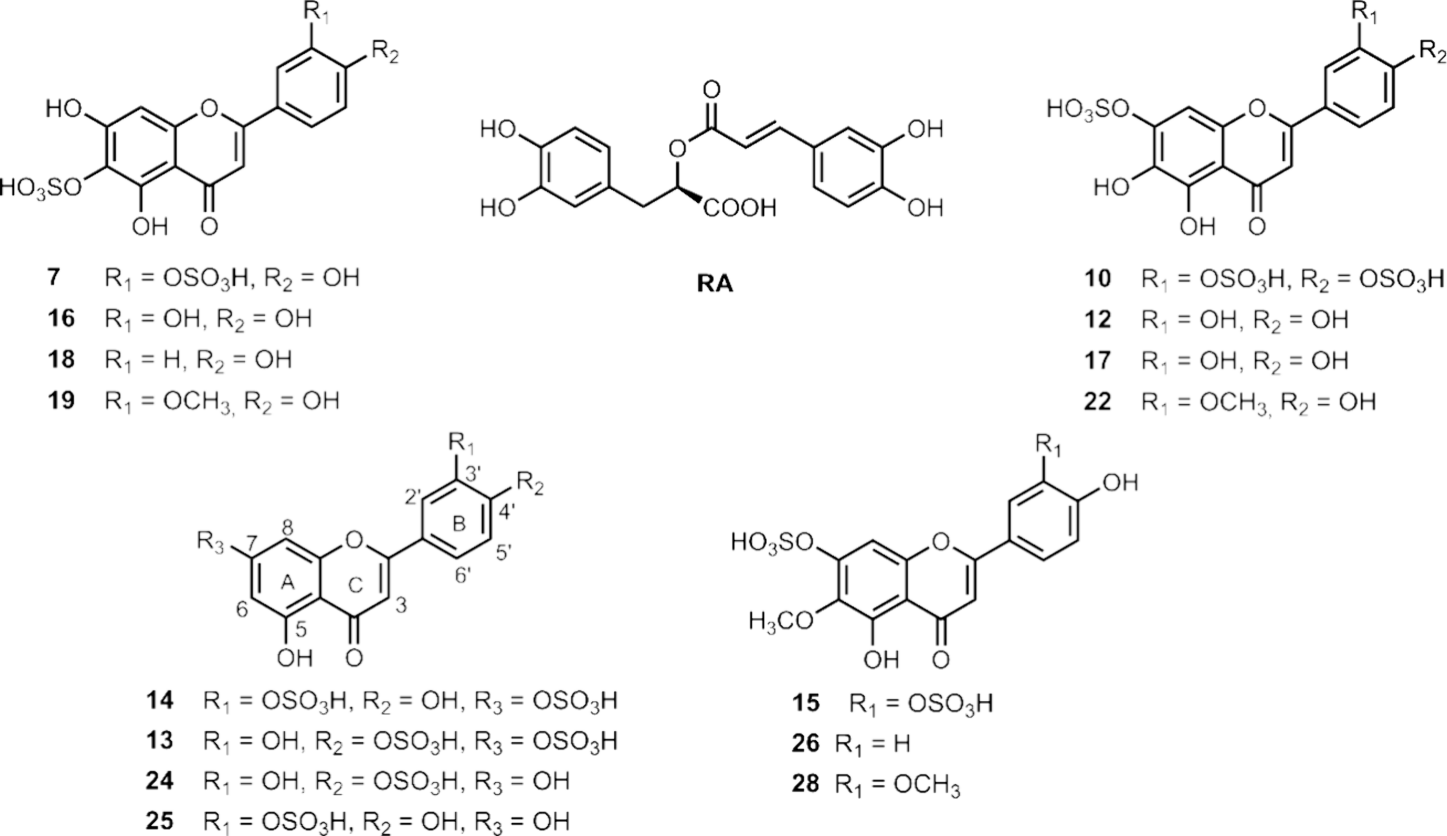
Structure of isolated compounds for *P. scouleri***7**, **10, 12–19**, **22**, **24**–**26**, **28** and **RA**.

The phytochemical in-depth study of *P. scouleri* was performed on the 2022 sample and began with the crude aqueous-methanolic extract of leaf tissue. By successive chromatography on C18 reverse-phase silica gel, six disulfate flavonoids (**7**, **10**, **12**-**15**), including a mixture of three (**7**, **10**, **12**), and nine monosulfated flavonoids (**16**-**19**, **22**, **24**-**26** and **28**) were isolated. Among these, compounds **7, 10, 12**, **18**, **19**, and **24** were previously undescribed, while compounds **13**, **16**, and **22** were reported for the first time in the *Phyllospadix* genus. The known phenolic acid, rosmarinic acid (**RA**), was also isolated (Figure 2). The identification of flavonoids and phenolic acid was achieved using 1D and 2D NMR data, MS, UV–vis, and comparison with literature data.

Compounds **7**, **10**, and **12** were isolated from the more polar fraction of the *P. scouleri* extract. The ^1^H NMR spectrum exhibited a complex set of signals in the δ_H_ 6 to 8 ppm range. Similarly, the ^13^C NMR spectrum displayed over 45 signals identified using the HMBC, HMQC, and COSY spectra. The mixture contained three disulfate flavonoids—compounds **7** and **12** exhibit an AMX spin system related to the ring B. The aromatic signals at 6.97 (1H, d, *J* = 8.5 Hz, H-5'), 7.70 (1H, dd, *J* = 2.4, 8.5 Hz, H-6'), and 7.92 (1H, dd, *J* = 2.3 Hz, H-2') belong to compound **7**, and at 6.96 (1H, d, *J* = 8.5 Hz, H-5'), 7.67 (1H, dd, *J* = 2.3, 8.4 Hz, H-6') and 7.88 (1H, dd, *J* = 8.5 Hz, H-2') belong to compound **12**. Compound **10** showed an ABC spin system related to the ring B at 7.50 (1H, d, *J* = 2.4 Hz, H-2'), 7.44 (1H, d, 8.3 Hz, H-5'), and 7.49 (1H, dd, *J* = 2.3, 8.3 Hz, H-6') respectively (Table 2). The chemical shift at the 2' position for **7** (δ_H_ 7.88, δ_C_ 120.4) and **12** (δ_H_ 7.93, δ_C_ 120.3) and the 5' position (δ_H_ 7.44, δ_C_ 121.9) for compound **10** suggests the presence of a sulfate group at the 3' position for compounds **7** and **12,** and the 4' position for compound **10** (Table 2).

**Table 2:** ^1^H and ^13^C NMR Chemical Shifts (δ in ppm) and Coupling Constants (*J* in Hz) for Flavonoid Disulfates **7**, **10**, **12** in DMSO-*d*_6_^a^

position	**7**		**10**		**12**	
	δ_H_	δ_*C*_	δ_H_	δ_*C*_	δ_H_	δ_*C*_
2		163.0, C		163.4, C		163.8, C
3	6.65, *s*	102.5, CH	6.82, *s*	103.8, CH	6.69, *s*	102.5, C
4		181.8, C		182.1, C		181.1, C
5		146.9, C		147.1, C		147.1, C
6		129.1, C		131.9, C		131.9, C
7		149.5, C		148.2, C		147.9, C
8	6.56, *s*	93.5, CH	7.29, *s*	98.4, CH	7.25, *s*	98.5, C
9		153.4, C		148.8, C		148.8, C
10		103.9, C		106.2, C		106.2, C
1'		120.9, C		126.1, C		121.5, C
2'	7.88, *d* (2.4)	120.4, CH	7.50, *d* (2.4)	119.5, CH	7.92, *d* (2.3)	120.3, C
3'		141.3, C		148.7, C		141.3, C
4'		152.6, C		144.6, C		152.2, C
5'	6.96, *d* (8.5)	117.4, CH	7.44, *d* (8.3)	121.9, CH	6.97, *d* (8.5)	117.4, CH
6'	7.67, *dd* (2.3; 8.4)	123.3, CH	7.49, *dd* (2.3; 8.3)	114.8, CH	7.70, *dd* (2.4; 8.5)	123.3, CH

a ^1^H recorded at 850 MHz and ^13^C recorded at 212.5 MHz.

**Figure 3:**
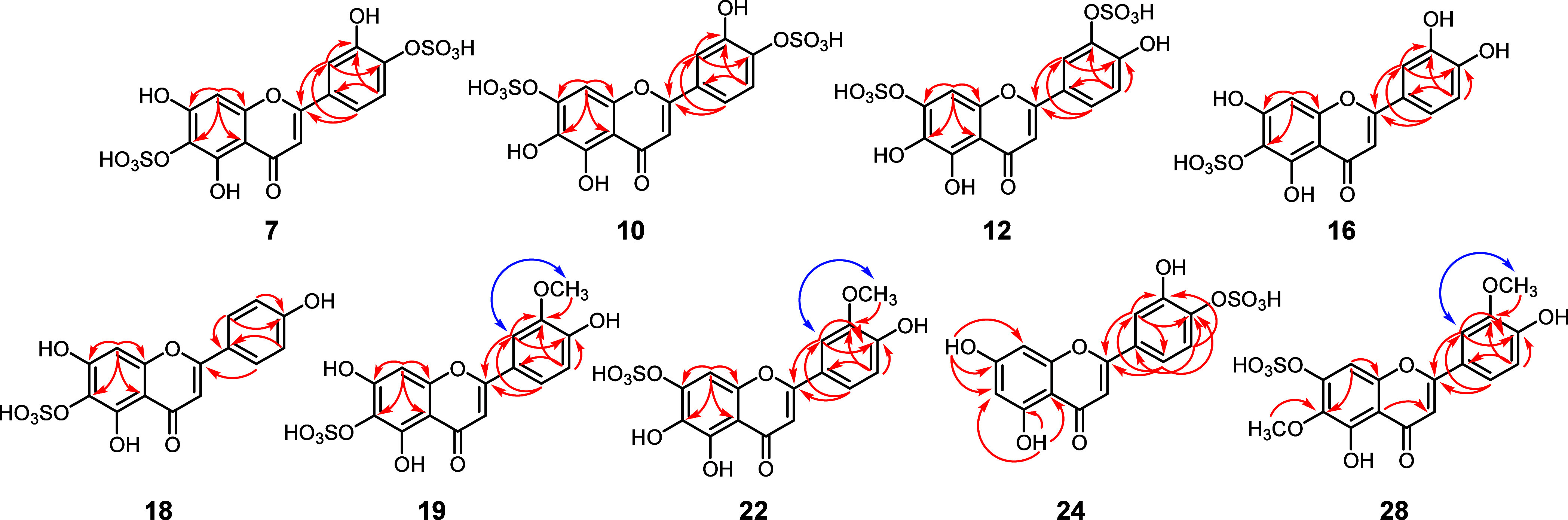
Key HMBC (red →) and NOESY (blue ↔) correlations of **7, 10, 12, 16, 18, 19, 22, 24** and **28.**.

Although the proton NMR spectra of individual flavonoids cannot be directly compared, the common diagnostic features for their 3'- and 4'-*O*-sulfates were also observed. Regioisomers can be differentiated by downfield-shifted H-2' and H-6' proton signals in 3'-*O*-sulfates and a downfield-shifted H-5' proton signal in 4'-*O*-sulfates.^25,28^ This also applies to carbon NMR spectra. In these spectra, all 4'-*O*-sulfates display a downfield shift for the C-3' carbon signal and an upfield shift for the C-4' signal relative to those for the parent compound. By contrast, a shift upfield of the C-3' carbon signal and a downfield shift for the C-4' signal was detected in the 3'-*O*-sulfates.^25,28^ The carbon and proton chemical shifts at positions 3' and 4' exhibited the typical *ipso* and *ortho* shifts due to a sulfate group at positions 3' and 4' in compounds **7**, **10**, and **12** (refer to Table 3). Compounds **7** and **10** showed displacement in ^1^H and ^13^C NMR of the environment in ring A. This confirms the overall substitution pattern of ring A, which is based on the long-range heteronuclear correlations from the aromatic methine H-8 (δ_H_ 7.29 for **10** and δ_H_ 7.26 for **12**) to C-7, C-9, C-6, and C-10 (Figure 3), with the sulfated group bonded to C-7 (Table 3). However, compound **7** bonds the sulfated group to C-6, and the chemical shift in ^1^H and ^13^C NMR indicates the overall substitution pattern of ring A. This is based on the long-range heteronuclear correlations from the aromatic methine H-8 (δ_H_ 6.56) to C-7, C-9, C-6, and C-10 (Table 2 and Figure 3).

**Table 3:** Diagnostic ^1^H and ^13^C NMR Sulfation Shifts for Flavonoid Sulfates **7**, **10**, **12**, **16-19**, **22**, **24**, **25**, and **28** (DMSO-*d*_6_); Values Are Expressed in ppm as δ (flavonoid sulfate) - δ (Flavonoid)

aglycon	sulfated derivatives	H-8	H-2'	H-5'	H-6'	C-6	C-7	C-8	C-2'	C-3'	C-4'	C-5'
6-hydroxyluteolin	6,3'-disulfate (**7**)	–0.02	+0.49	+0.08	+0.26	–1.2	–4.4	–0.9	+6.5	–5.1	+2.3	+0.8
	7,4'-disulfate (**10**)	+0.75	+0.11	+0.56	+0.08	+2.1	–5.7	+4.0	+5.6	+2.3	–5.7	+5.4
	7,3'-disulfate (**12**)	+0.71	+0.53	+0.09	+0.29	+2.1	–6.0	+4.1	+6.4	–5.1	+1.9	+0.8
	6-sulfate (**16**)	–0.01	–0.01	–0.01	–0.02	–1.3	–4.3	–0.8	–0.7	–0.8	–0.9	–0.7
	7-sulfate (**17**)	+0.73	+0.06	–0.01	+0.02		–5.9	+3.8	–0.6	–0.8	–0.7	–0.4
scutellarein(6-OH)	6-sulfate (**18**)	–0.05	–0.02	–0.06	–0.02	–1.3	–4.3	–0.7	–0.2	–0.7	–0.2	–0.7
nodifloretin(6-OH)	6-sulfate (**19**)	+0.06	–0.01	–0.02	–0.09	–1.2	–4.3	–0.6	–0.1	0	–0.1	0
	7-sulfate (**22**)	+0.74	–0.02	–0.03	–0.05	+2.2	–5.7	+4.2	–0.2	0	–0.1	+0.1
luteolin	4'-sulfate (**24**)	+0.05	+0.08	+0.56	+0.07	0	0	+0.2	+1.2	+3.1	–5.3	+6.0
	3'-sulfate (**25**)	+0.06	+0.51	0	+0.29							
jaceosidin(6-OMe)	7-sulfate (**28**)	+0.66	–0.07	–0.09	–0.04	+2.8		+4.1	–0.4	–0.1	+0.2	0

High-resolution LC-MS was used to confirm the presence of the *O*-sulfate group in the mixture. Compound **7** had an observed accurate mass (molecular formula) of *m*/*z* 463.9640 (C_15_H_11_O_13_S_2_). The accurate masses for compounds **10** and **12** as [M + H]^+^ ion were *m*/*z* 463.9642 (C_15_H_11_O_13_S_2_) and *m*/*z* 463.9640 (C_15_H_11_O_13_S_2_), respectively. The disulfate substitution for **7, 10,** and **12** were additionally confirmed by significant ions at *m*/*z* 383 [M-80 + H]^+^ and *m*/*z* 303 [M-160 + H]^+^ observed for all three compounds. The structures of this mixture of regio-isomers of disulfate flavonoids were defined by spectroscopic and HR-MS data as 6-hydroxyluteolin 6,3'-disulfate (**7**), 6-hydroxyluteolin 7,4'-disulfate (**10**) and 6-hydroxyluteolin 7,3'-disulfate (**12**), as shown in Figure 2. The **7**, **10**, and **12**- structures were confirmed as undescribed products by analyzing the 2D NMR data from the HSQC and HMBC spectra and querying the SciFinder database.

Compound **19** was isolated as a yellow amorphous solid after elution at 31.8 min. The HR-ESI-MS displayed a quasi-molecular ion peak at *m*/*z* 397.0230 [M + H]^+^, suggesting a pseudo molecular formula of C_16_H_13_O_10_S (calcd.; 397.0229). The presence of one sulfate group was confirmed by another significant ion at *m*/*z* = 317 [M-80+H]^+^. The ^1^H NMR spectrum recorded in DMSO-*d*_6_ showed three aromatic signals related to the B-ring: 6.92 (1H, d, *J* = 8.8 Hz) for H-5', 7.55 (1H, d, *J* = 2.2 Hz) for H-2', and 7.54 (1H, dd, *J* = 2.2, 8.3 Hz) for H-6' (Table 4). The ^13^C NMR spectrum, in conjunction with the HSQC spectrum, revealed the presence of a carbonyl (δ_C_ 182.0), five aromatic methines (δ_C_ 93.8, 102.5, 110.0, 115.5, and 120.1), a methoxy (δ_C_ 55.8), seven tertiary carbons all oxygenated (δ_C_ 163.4, 153.2, 150.5, 149.6, 147.9, 146.9 and 129.1), and two aromatics quaternary carbons (δ_C_ 121.7 and 103.9) (Table 5). The presence of methoxy substituent located at C-3' was confirmed by the long-range heteronuclear correlation between the protons at δ_H_ 3.88 and C-3' (δ_C_ 147.9), as well as through 2D NOESY NMR correlations, which demonstrated the proximity of protons from the methoxy group with the hydrogen-2' belonging to ring B (δ_H_ 7.55) (see Figure 3). The ^1^H and COSY data indicated the lack of coupling for ring A. The chemical shift of the aromatic methine (δ_H_ 6.60, δ_C_ 93.8) indicated its location between one oxygen substituent and the quaternary carbon belonging to the heterocyclic ring C, placing this group at C-8,^28^ and leaving the phenolic hydroxy groups to be assigned at C-5 and C-7. The sulfate moiety can only be located at C-6 due to the chemical shift for the C-6 (δ_C_ 129.1) and the absence of a hydroxy or methoxy group. A sulfate ester group typically causes upfield displacement for the *ipso* carbon and downfield displacement for the *ortho* and *para* related carbon, as Agrawal^28^ and Barron et al.^25^ reported. However, we observed an opposite chemical shift for carbons in the ortho position to C-6. Both C-7 and C-5 showed upfield displacement (δ_C7_ 149.6; δ_C5_ 146.9) when compared to aglycones, such as 6-hydroxyluteolin or nodifloretin, which exhibit this substitution pattern (C5-OH, C6-OH_,_ and C7-OH) in ring A, lacking the sulfate moiety in C-6 (Table 3).^28^ The shielding of C-5 and C-7 may be due to steric compression exerted by the sulfate group in the C-6 of the aromatic ring. Long-range heteronuclear correlations confirmed ring A’s substitution pattern from the aromatic methine H-8 (δ_H_ 6.60) to C-7, C-9, C-6, and C-10 (Figure 3). This spectroscopic data defines the structure of nodifloretin 6-sulfate (**19**), as shown in Figure 2. The analysis of the 2D NMR data from the HSQC, HMBC, and NOESY spectra and querying of the SciFinder database confirmed that structure **19** is an undescribed product.

**Table 4:** ^1^H-NMR Chemical Shifts (δ in ppm) and Coupling Constants (*J* in Hz) for Flavonoid Sulfates **16**-**19**, **22**, **24**, **25**, and **28** in DMSO-*d*_6_^a^

position	**16**	**17**	**18**	**19**	**22**	**24**	**25**	**28**
3	6.62, *s*	6.70, *s*	6.73, *s*	6.85, *s*	6.91, *s*	6.80, *s*	6.70, *s*	6.96, *s*
6						6.20, *d* (2.0)	6.20, *d* (2.0)	
8	6.53, *s*	7.27, *s*	6.57, *s*	6.60, *s*	7.28, *s*	6.49, *d* (2.0)	6.49, *d* (2.0)	7.35, *s*
2'	7.38, *d* (2.3)	7.45, *d* (2.4)	7.91, *d* (9.0)	7.55, *d* (8.2)	7.56, *d* (2.2)	7.47, *d* (2.3)	7.90, *d* (2.5)	7.56, *d* (2.2)
3'			6.91, *d* (8.7)					
5'	6.87, *d* (8.3)	6.87, *d* (8.4)	6.91, *d* (8.7)	6.92, *d* (2.2)	6.91, *d* (8.2)	7.44, *d* (8.4)	6.88, *d* (8.4)	6.93, *d* (8.3)
6'	7.39, *dd* (2.3; 8.3)	7.43, *dd* (2.4; 8.4)	7.91, *d* (9.0)	7.54, *dd* (2.2; 8.2)	7.58, *dd* (2.2; 8.2)	7.48, *dd* (2.3, 8.4)	7.69, *dd* (2.3, 8.4)	7.59, *dd* (2.2, 8.3)
OMe-6								3.75, *s*
OMe-3'				3.88, *s*	3.90, *s*			3.90, *s*
5-OH						10.85, *s*		
7-OH						12.90, *s*		
3'–OH						9.30, *s*		

a Recorded at 850 MHz.

**Table 5:** ^13^C-NMR Chemical Shifts (δ in ppm) for fLavonoid Sulfates, **16**-**19**, **22**, **24** and **28** in DMSO-*d*_6_^a^

position	**16**	**17**	**18**	**19**	**22**	**24**	**28**
2	163.5, C	163.9, C	163.4, C	163.4, C	164.1, C	163.2, C	164.9, C
3	102.2, CH	102.2, CH	102.7, CH	102.5, CH	102.6, CH	104.2, CH	102.9, CH
4	181.8, C		181.9, C	182.0, C	182.3, C	181.7, C	182.1, C
5	146.9, C		147.0, C	146.9, C	147.2, C	161.4, C	
6	129.0, C		129.0, C	129.1, C	132.0, C	98.8, CH	134.1, C
7	149.6, C	148.0, C	149.6, C	149.6, C	148.2, C	164.1, C	
8	93.6, CH	98.2, CH	93.7, CH	93.8, CH	98.6, CH	93.9, CH	98.3, CH
9	153.2, C		153.2, C	153.2, C	147.8, C	151.3, C	151.8, C
10	103.6, C	106.1, C	103.9, C	103.9, C	106.7, C	103.7, C	106.3, C
1'	121.2, C	121.3, C	121.4, C	121.7, C	121.6, C	126.1, C	121.6, C
2'	113.2, CH	113.3, CH	128.8, CH	110.0, CH	109.9, CH	114.5, CH	109.9, CH
3'	145.6, C	145.6, C	115.9, CH	147.9, C	147.9, C	148.7, C	147.9, C
4'	149.4, C	149.5, C	160.9, C	150.5, C	150.5, C	144.3, C	150.9, C
5'	115.9, CH	116.2, CH	115.9, CH	115.6, CH	115.7, CH	121.9, CH	115.7, CH
6'	118.7, CH	119.4, CH	128.8, CH	120.1, CH	120.3, CH	120.1, CH	120.4, CH
OMe-6							60.3, CH_3_
OMe-3'				55.8, CH_3_	55.9, CH_3_		55.6, CH_3_

a Recorded at 212.5 MHz.

Compound **22** was isolated as a yellow amorphous solid after elution at 33.3 min. The negative ESI-MS spectrum displayed a quasimolecular peak [M-H]^−^ at 397 *m*/*z*, which was compatible with the molecular formula of C_16_H_12_O_10_S. Another significant ion at 317 *m*/*z* [M-80 + H]^+^ confirms the presence of one sulfate group, indicating that **22** is an isomer of **19**. However, the NMR data revealed some significant structural differences between the two compounds, including a sulfate group at the 7-position based on the hydrogen and carbon resonance of the methine at the 8-position (δ_H_ 7.28, δ_C_ 98.6). H-8 showed a strong deshielded environment (Table 3) compared to the methine-8 of compound **19** bearing the sulfate group at position 6 (Figure 2). The chemical shifts in the ^1^H and ^13^C NMR spectra for the environment in ring C were similar to those observed in compound **19** (Tables 4 and 5). The NOESY correlation between the methoxy hydrogen and H-2' confirms the presence of the methoxy group in position-3' (as shown in Figure 3). The observed long-range heteronuclear correlations also confirm the presence of the methoxy group in position-3' and the general substitution pattern of the B ring in the HMBC spectrum (Figure 3). Additionally, the overall substitution pattern of ring A was confirmed by the long-range heteronuclear correlations from the aromatic methine H-8 (δ_H_ 7.28) to C-7, C-9, C-6, and C-10 (Figure 3), with the sulfated group bonded to C-7 (Table 3). These spectroscopic data defined the structure of nodifloretin 7-sulfate (**22**), as displayed in Figure 2. Analysis of the HSQC, HMBC, and NOESY spectra confirmed the structure of compound **22**. This compound has been previously identified in the aerial parts of the maritime growing plant *Lippia nodiflora*,^29^ but no NMR data has been reported until now.

Compound **18**, eluted at 28.2 min, was isolated as a yellow amorphous solid. The HR-ESI-MS displayed a quasi-molecular ion peak at *m*/*z* 367.0135 [M + H]^+^, suggesting a pseudo molecular formula of C_15_H_11_O_9_S (calcd.; 367.0124). The presence of one sulfate group was confirmed by another significant ion at *m*/*z* = 287 [M-80+H]^+^ confirmed. The ^1^H NMR spectrum recorded in DMSO-*d*_6_ revealed an AA'BB' spin system comprising two pairs of two-proton doublets at δ_H_ 7.91 (2H, d, *J* = 9.0 Hz, H-2'/6') and 6.91 (2H, d, *J* = 8.7 Hz, H-3'/5'), defining the B ring as a *para* disubstituted aromatic ring (Table 4). The presence of the hydroxy substituent was confirmed by the long-range heteronuclear correlations between the phenolic protons H-2'/6' and H-3'/5' at 7.91 and 6.91 ppm with C-4' (δ_C_ 160.9), as illustrated in Figure 3. The ^13^C NMR spectrum, in combination with the HSQC spectrum, revealed the presence of a carbonyl (δ_C_ 181.9), six aromatic methines (δ_C_ 93,7, 102.7, and with two carbon each, 128,8, and 115.9), six tertiary carbons all oxygenated (δ_C_ 163.4, 160.9, 153.2, 149.6, 147.0 and 129.0), and two aromatic quaternary carbons (δ_C_ 121.4 and 103.9) (Table 5). The displacement in ^1^H and ^13^C NMR of the environment in ring A was similar to that of compound **19** (Tables 3-5), including the overall substitution pattern of ring A based on the long-range heteronuclear correlations from the aromatic methine H-8 (δ_H_ 6.57) to C-7, C-9, C-6, and C-10 (Figure 3), with the sulfated group bonded to C-6 (Table 3). These spectroscopic data define the structure of scutellarein 6-sulfate (**18**), as displayed in Figure 2. The analysis of the 2D NMR data from the HSQC and HMBC spectra confirmed that structure **18** is a previously undescribed product, which was also verified by querying the SciFinder database.

Compound **16** was isolated as a yellow amorphous solid after elution at 27.2 min. The HR-ESI-MS displayed a quasi-molecular ion peak at *m*/*z* 383.0081 [M + H]^+^, which suggested a pseudomolecular formula of C_15_H_11_O_10_S (calcd.; 383.0083). Another significant ion at *m*/*z* = 303 [M-80 + H]^+^ confirmed the presence of one sulfate group. The ^1^H NMR spectrum recorded in DMSO-*d*_6_ showed aromatic signals at 6.87 (1H, d, *J* = 8.3 Hz), 7.38 (1H, d, *J* = 2.3 Hz), 7.39 (1H, dd, *J* = 2.3, 8.3 Hz) related to the B-ring (H-5', H-2' and H-6' respectively) (refer to Table 4). The NMR data revealed a notable structural difference between compounds **19** and **16**. This difference is due to substituting the methoxy group with a hydroxy group at the C-3' carbon of ring B and the lack of the methoxy group signal. The HMBC correlations confirmed the hydroxy group at C-3' (Figure 3). The chemical shifts in the ^1^H and ^13^C NMR spectra for the environment in ring A were similar to those of compounds **18** and **19** (Tables 3-5). This includes the overall substitution pattern of ring A, as evidenced by the long-range heteronuclear correlations from the aromatic methine H-8 (δ_H_ 6.53) to C-7, C-9, C-6, and C-10 with the sulfated group bonded to C-6 (Figure 3 and Table 3). These spectroscopic data define the structure of 6-hydroxyluteolin 6-sulfate (**16**), as displayed in Figure 3. The structure was confirmed by analyzing the HSQC and HMBC spectra, which confirmed the structure of **16**. Similarly to compound **22**, compound **16** has previously been found in the maritime growing *L. nodiflora*,^29^ yet no NMR data has been published.

Compound **17**, eluted at 28.0 min, was isolated as a yellow amorphous solid. The negative ESI-MS spectrum displayed a quasimolecular peak [M-H]^−^ at 381 *m*/*z*, which was compatible with the molecular formula of C_15_H_10_O_10_S. The ion at 301 *m*/*z* [M-80 – H]^−^ confirms the presence of one sulfate group, indicating that **17** is an isomer of **16**. NMR data revealed considerable structural differences between these two compounds, specifically in ring A, in which compound **17** possesses a sulfate group at the 7-position based on the hydrogen and carbon resonance of the methine at the 8-position (δ_H_ = 7.27, δ_C_ = 98.2) showing a strong deshielded environment (Table 3) compared to the methine-8 of compound **16** bearing the sulfate group at position 6 (Figure 2). The displacement in ^1^H and ^13^C NMR of the environment in ring B was similar to compound **16** (Tables 4 and 5). These spectroscopic data defined the structure of 6-hydroxyluteolin 7-sulfate (**17**), as shown in Figure 2. This compound was previously found in *P. torreyi*(12) and the maritime growing *L. nodiflora*,^29^ though NMR data has not yet been reported.

Compound **24**, eluted at 34.2 min, was isolated as a yellow amorphous solid. The ^13^C NMR spectrum in DMSO-*d*_6_, in conjunction with the HSQC spectrum, revealed the presence of a carbonyl (δ_C_ 181.7), six aromatic methines (δ_C_ 93.9, 98.8, 104.2, 114.5, 117.1, and 121.9), six tertiary carbons all oxygenated (δ_C_ 164.3, 163.2, 161.4, 151.3, 148.7, and 144.3), and two aromatic quaternary carbons (δ_C_ 126.1 and 103.7) (Table 5). The ^1^H NMR spectrum displays six proton signals in the aromatic region, consistent with a luteolin derivative. This includes a pair of *meta* coupled protons at δ_H_ 6.20 (1H, d, *J* = 2.2 Hz, H-6) and δ 6.49 (1H, d, *J* = 2.2 Hz, H-8), as well as a proton singlet at δ_H_ 6.80 (H-3) (Table 4). The hydrogen-bonded hydroxy group at δ_H_ 12.90 was located at C-5, as confirmed by its HMBC correlations to C-10 (δ_C_ 103.7), C-6 (δ_C_ 98.8), and C-5 (δ_C_ 161.4). The HMBC correlation between the protons at δ_H_ 10.85 to C-8 (δ_C_ 93.9), C-6 (δ_C_ 98.8), and C-7 (δ_C_ 164.13), located in the other hydroxy group was positioned at C-7 (Figure 3 and Tables 4-5). The other aromatic proton belongs to the ABC spin system at 7.44 (1H, d, *J* = 8.4 Hz), 7.47 (1H, d, *J* = 2.3 Hz), 7.48 (1H, dd, *J* = 2.3, 8.4 Hz) related to the B-ring (H-5', H-2' and H-6' respectively) (Table 5). Although the proton NMR spectra of individual flavonoids were not directly comparable, the common diagnostic features for their 3'- and 4'-*O*-sulfates were also observed. Since 3'-*O*-sulfated compounds showed in their NMR data a downfield shift at the positions 2' and 6', as well as 5' in 4'-*O*-sulfates compounds.^25,28^ The chemical shift for carbon in position 4' showed the typical *ipso* and *ortho* shifts due to the presence of a sulfate group at position 4' (strong deshielding of H-5', C-5' and C-3'; shielding of C-4') (Table 3). The HMBC spectrum confirmed the general substitution pattern of the B ring with observed long-range heteronuclear correlations (Figure 3). After NMR analyses, the isolated fraction containing minor amounts of **24** was subjected HR-ESI MS analysis. The fraction only contained the aglycone at *m*/*z* = 287.0550 [M-80+H]^+^ due to, desulfation after storage. This spectroscopic data defined the structure of luteolin 4'-sulfate (**24**), as displayed in Figure 3 previously synthesized by Káňová et al.^30^ To our knowledge, this compound has not been previously identified as a natural product.

Compound **25**, which eluted at 27.9 min, is an isomer of **24** and was isolated as a yellow amorphous solid. The NMR data revealed some significant structural differences between the isomers, particularly in Ring B. Compound **25** shows an AMX spin system related to this ring at 6.88 (1H, d, *J* = 8.4 Hz, H-5'), 7.69 (1H, dd, *J* = 2.3, 8.4 Hz, H-6'), and 7.90 (1H, dd, *J* = 2.5 Hz, H-2') respectively (Table 4), whereas compound **24** which exhibits an ABC spin system in the B ring. The sulfate group is attached to the 3'-position, as confirmed by the strong deprotection shown in the H-2' and H-6' proton signals (Table 4) characteristic of 3'-*O*-sulfates. This contrasted with compound **24**, which has the sulfate group in position 4, showing an H-5' proton signal displaced downfield characteristic of 4'-*O*-sulfates (Table 3). Comparison with literature data confirmed the identification of luteolin 3'-sulfate (**25**), as shown in Figure 2. This compound has previously been found in *P. torreyi*,^12^*Zostera marina*,^31^*Z. noltei*,^26^ and *Z. asiatica*.^32^

Compound **28,** eluted at 38.7 min, was isolated as a yellow amorphous solid. The negative ESI-MS spectrum displayed a quasimolecular peak [M-H]^−^ at 409 *m*/*z*, which was compatible with the molecular formula of C_17_H_14_O_10_S. Also, the ion at 329 *m*/*z* [M-80 – H]^−^ confirms the presence of a sulfate group. The ^1^H NMR spectrum showed two singles (2 × 3H) at δ_H_ 3.75 and 3.90 and five proton signals at 6.93 (1H, d, *J* = 8.3 Hz, H-5'), 6.96 (1H, s, H-3), 7.35 (1H, s, H-8), 7.56 (1H, d, *J* = 2.2 Hz, H-2'), 7.59 (1H, dd, *J* = 2.2, 8.3 Hz, H-6') (Table 3). The aromatic signal reveals an AMX spin system related to ring B. Of the two methoxy groups confirmed by HMBC correlations, one is located in ring B, at position 3', as shown by the correlation between the ^1^H signal at 3.75 ppm and C-3' at 147.9 ppm, as well as by 2D NOESY NMR correlations between proton of the methoxy group and the hydrogen-2' (δ_H_ 7.56) (Figure 3 and Tables 4 and 5). The second methoxy in ring A was confirmed at position C-6 by the long-range heteronuclear correlation between the protons at δ_H_ 3.90 and C-3' (δ_C_ 134.1) (Figure 3 and Tables 4 and 5). Carbon and proton resonances showed the typical shifts due to the presence of a sulfate group at position 7 (strong deshielding of H-8, C-8, and C-6; Barron et al.^25^) (Table 3). In addition, the general substitution pattern of the B ring is confirmed by the observed long-range heteronuclear correlations in the HMBC spectrum (Figure 3). These spectroscopic data define the structure of jaceosidin 7-sulfate (**28**), as shown in Figure 2, which was previously found in *P. torreyi*,^12^ and the maritime growing *L. nodiflora*.^29^ However, no NMR data have been reported for this compound before.

The compounds **15** (nepetin 7,3'-disulfate), RA (rosmarinic acid), and 26 (hispidulin 7-sulfate) were identified based on a comparison of their UV–vis, MS and NMR spectral data with previously published data.^12,32,33^ (Figure 2). All these compounds have been documented in *P. torreyi*.^12^

The minor Compounds **13** and **14** were suspected to be structural isomers of the other isolated flavonoids. The polarity of these compounds is higher compared to nepetin 7,3'-disulfate (**15**), however less polar than the mixture of 6-hydroxyluteolin disulfates (**7**, **10**, **12**) (Figure 1), leading us to identify them as disulfated derivatives. The ^1^H NMR spectrum showed six proton signals in the aromatic region, consistent with luteolin derivatives. UV spectral studies of several naturally occurring and synthetic sulfated compounds indicated that sulfation at ring B induces a significant hypsochromic shift in Band I due to the electron-withdrawing effect of the sulfate group.^25^ In contrast, sulfation at ring A does not significantly affect UV absorption. In the case of luteolin, the Band I UV (λ max) values were as follows (nm): 7,4'-disulfate (320)^25^ 7,3'-disulfate (335).^12^ Our results with luteolin derivatives (DAD online) show the same tendency: 7,3'-disulfate (335) and 7,4'-disulfate (320) (Table 1). From these values, we tentatively assign compound **13** as luteolin 7,4'-disulfate and compound **14** as luteolin 7,3'-disulfate, as displayed in Figure 2. Compound **13** was previously reported in *P. torreyi,*(12) while compound **14** was identified *Z. marina*.^26^

The aglycones identified in this study corroborate previous findings on the distinctive pattern in the *Phyllospadix* genus.^12−15^ This pattern is characterized by a high prevalence of 6-hydroxy (*P. scouleri*) and 5- or 6-methoxy-substituted (*P. torreyi*) aglycones, notably absent in the *Zostera* species. To our knowledge, another example found in seagrass is the 6-hydroxyflavonoid scutellarein 7-glucoside found in the tropical seagrass *Halophila johnsonii* (*Hydrocharitaceae*).^34^ The nodifloretin aglycone (**22**) identified in *P. scouleri* is a novel discovery for seagrasses, as it has not been previously reported in other seagrass species. It has only been tentatively identified in the plant *L. nodiflora,* that grows in a maritime environment.^29^

Generally, most sulfated flavonoids detected in *P. torreyi* and *P. scouleri* are substituted at the 7-position, corresponding to the most reactive hydroxyl groups in flavones (7-OH). In *P. torreyi*, seven out of 12 flavonoids (85%) have a sulfate group at position 7. Conversely, in *P. scouleri*, nine out of 17 flavonoids (57%) feature a sulfate group at the same position. These findings align with the patterns documented by Grignon-Dubois et al. for *P. torreyi*,^12^ where 11 out of 13 sulfated flavonoids have a sulfate group at position 7. Additionally, four out of 15 isolated flavonoids from *P. scouleri* contain a sulfate group at position 6, accounting for 18.6% of all sulfated flavonoids. In contrast, only one flavonoid in *P. torreyi* is sulfated at position 6, representing a mere 0.4% of its total flavonoid content. Interestingly, Grignon-Dubois et al. previously reported no flavonoids with sulfate groups at position 6 in *P. torreyi*.^12^

As previously mentioned, the sulfated flavonoids in *P. torreyi* are predominantly disulfated, accounting for 84.3% of the total. This contrasts with *P. scouleri*, where disulfated flavonoids comprise only 25.2%. In the *Zostera* genus, disulfated flavonoids are found in *Z. marina* (24%) but are absent in *Z. noltei*.^26,27^ Glycosylation is not commonly observed in seagrasses, although monoglycosylation occurs in several families. In the current study, sulfated glycosides and diglycosides were tentatively identified in both *P. scouleri* (**1**-**4**) and *P. torreyi* (**2**, **3**, **5**, and **6**) for the first time. However, the small quantities only allowed for tentative LC-MS identification. Besides our findings, diglycosylation has previously been documented only in the Egyptian seagrass *Thalassodendron ciliatum*.^35^

### Potential Ecological Implications

The metabolic functional roles of flavonoid sulfates in seagrasses are still unclear.^1^ The phenolic chemistry of *Z. marina*, including sulfated flavonoids, has been assessed concerning its adaption/acclimation to environmental gradient associated with latitude, depth, and even plant tissue position within eelgrass meadows.^36^ Grignon-Dubois and Rezzonico^37^ further analyzed the phenolic content of *Z. marina* populations across four bioregions, finding geographic variation in concentration but a consistent presence of rosmarinic acid and luteolin 7,3′-disulfate as dominant compounds. They also documented intraspecific flavonoid chemotypes of *Z. noltei* across its broad geographical distribution in the Mediterranean and NE Atlantic coast.^27^ In the case of surfgrasses, changes in total phenols of *P. torreyi* and *P. scouleri* from Baja California (Mexico) have been related to their acclimation and stress responses to emersion^16^ and marine heatwaves.^22,38^ The stable high concentration of phenolic compounds has been linked to the antioxidant defense of *P. torreyi* facing emersion during low tides.^16^ In *P. scouleri*, marine heatwaves have led to a decline in phenolic content and antioxidant responses, which in turn has been linked to plant metabolic disruption and oxidative damage.^38^ Although our results represent an important advance in the knowledge of the sulfated flavonoid profiles of surfgrasses, further research is needed to better understand their biosynthesis, accumulation dynamics, and physiological/ecological functions. *Phyllospadix* is considered one of the most divergent seagrass genera due to its unique physical habitat and adaptive physiological and morphological characteristics.^10^ Further investigation is needed to determine how various spatial and temporal environmental conditions affect flavonoid sulfate biochemistry. Additionally, hybridization between *P. torreyi* and *P. scouleri* can occur,^39−41^ potentially resulting in different chemotypes. However, this and other potential sources of variation in the phenolic profiles of surfgrasses remain unknown. For instance, the microbiome associated with seagrasses can induce chemical changes in the host plant,^42^ potentially influencing its phenolic compounds.

## Conclusions

This study provides the first comprehensive analysis of flavonoids in *P. scouleri*, identifying one phenolic acid (rosmarinic acid) and 15 sulfated flavonoids (**7**, **10**, **12**–**19**, **22**, **24**–**26**, **28**) from aqueous-methanolic foliar extracts. Six were previously undescribed (**7**, **10**, **12**, **18**, **19**, and **24**), and three (**13**, **16**, and **22**) are newly reported for the genus *Phyllospadix*. The flavonoid profile of *P. scouleri* was dominated by 6-hydroxyflavonoids, comprising 70% of its total flavonoid content. A comparative analysis with *P. torreyi* revealed similar flavonoid quantities in samples collected from nearby areas in May 2024: *P. scouleri* (20.03 ± 0.23 mg) and *P. torreyi* (20.67 ± 0.15 mg). *P. scouleri* contained 25.2% disulfated and 66.5% monosulfated flavonoids, whereas *P. torreyi* showed a predominance of disulfated flavonoids (84.3%) over monosulfated ones (11.9%). Both species had comparable ratios of total flavonoids to rosmarinic (∼6:1). Additionally, sulfated glycosides were tentatively identified in *P. scouleri* (**1**-**4**) and *P. torreyi* (**2**, **3**, **5**, and **6**) for the first time. Flavonoid sulfates often co-occur with flavonoid glycosides, as observed in the genus *Zostera*, which is closely related to *Phyllospadix*.^10,25−27^ Notably, our findings differ from previous studies on *P. torreyi* flavonoid sulfates by Grignon-Dubois et al.,^12^ likely because our specimens were collected 150 km further south from intertidal and subtidal zones. These regional and habitat-based differences suggest that environmental factors may drive ecotypic or genotypic adaptations in flavonoid sulfate biosynthesis, possibly as a response to local ecological stresses.^5,10^ Future research should explore the habitat-specific biosynthetic pathways of these compounds across various environmental conditions to deepen our understanding of the ecological function of sulfated flavonoids in *Phyllospadix*.

## Experimental Section

### General Experimental Procedures

Formic acid (FA) and all the solvents used (HPLC-grade) were purchased from Aldrich Chemical Co. (Saint-Louis, Missouri, USA).

The Agilent 1100 HPLC was used for Analytical HPLC. It was equipped with an Agilent 1200 series diode array detector and a 250 × 4.6 mm inside diameter, 5 μm ODS Hypersil column (Agilent Technologies). Elution was performed using two solvents, (A) water (0.5% FA and (B) methanol (0.5% FA). The analytical HPLC gradient used was: 0–35 min; 10% - 75% B, 35–36 min; 75% - 100% B, isocratic elution 36–45 min; 100% B, 45–46 min 100% - 10% B and finally isocratic 46–48 min 10% B. The flow rate was 1.0 mL/min, and 20 μL aliquots were injected using an Agilent 1100 series microautosampler. The UV–vis absorption spectra were recorded online during HPLC analysis over the wavelength range of 240–600 nm in steps of 2 nm increments. Preparative HPLC: a Gilson 321 pump equipped with an Ultimate 3000 variable wavelength detector, a 25 × 2.2 cm (10 μm) Econosphere C18 column (Grace, Deerfield, IL), and the solvents (A) water (0.1% FA) and (B) acetonitrile (0.1% FA). The following gradient was employed: 0–5 min; 5% - 10% B, 5–25 min; 10% - 30% B, 35–45 min; 30% - 40% B, 45–60 min 50% B. The flow rate was 10 mL/min.

All NMR spectra (^1^H, ^13^C, and 2D) were recorded at 850 MHz at 25 °C on a Bruker 850 MHz instrument equipped with a ^1^H, ^13^C, ^15^N triple resonance cryogenic probe. Chemical shifts are expressed in δ (*ppm*). Samples were dissolved (0.2 mL) in (DMSO-*d*_6_). The residual solvent peaks specific to the deuterated dimethyl sulfoxide (DMSO-*d*_6_) solvent was used as an internal reference; DMSO-*d*_6_: 2.50 ppm (^1^H NMR) and 39.52 ppm (^13^C NMR). The following abbreviations were used to describe the multiplicities: br = broad, s = singlet, d = doublet, dd = doublet of doublets, ddd = doublet of doublets of doublets, t = triplet, q = quartet, m = multiplet, coupling constants in Hz and integration.

Low-resolution liquid chromatography–mass spectrometry (LC-ESI MS(Q); LR LC-MS) was performed using an Agilent Technologies 1260 Infinity Series system and an Agilent Technologies 6420A triple quadrupole mass spectrometry detector. The ionization modes were negative and positive, and the capillary voltage was set to 3000 V. The gas temperature was maintained at 300 °C, and the gas flow rate was set to 3.0 L/min. The acquisition range was 100–1000 *m*/*z*. The HPLC elution profile for HPLC began with 90% A (water with 0.1% formic acid) and 10% B (acetonitrile with 0.1% formic acid). This was followed by an isocratic elution from 0 to 2 min, and then a linear gradient elution to 50% B (2–15 min). Separation was performed using a 50 × 4.6 mm internal diameter, 1.8 μm Agilent Zorbax Eclipse XDB C18 column. The LC column temperature was maintained at 25 °C.

The High-resolution liquid chromatography–mass spectrometry (LC-ESI MS(Q-TOF); HR LC-MS) was performed using UPLC analyses on an ACQUITY UPLC I Class system connected to a Synapt G2-S mass spectrometry detector (Waters Corporation, Milford, USA) with positive electrospray ionization sources (ESI+). A concentration of 200 ng/mL of leucine enkephalin was used as a Lockmass at a flow rate of 10 μL/min to allow for the correction of exact mass measurements. The ionization mode was set to positive, and the capillary voltage, the gas temperature, and the gas flow rate were adjusted to 2500 V, 350 °C, and 13 L/min, respectively. The LC column and solvent system used were similar to those in LR LC-MS, with a flow rate of 0.3 mL/min and an injected volume of 2 μL. The elution profile for HR-LC consisted of initial conditions of 90% A. Isocratic elution was performed from 0 to 2 min, followed by a linear gradient elution to 50% B (2–15 min), isocratic elution 50% B (15–15.5 min), linear gradient 100% B (15.5–17 min), linear elution 10%B (17–17.5 min) and isocratic elution 10% B (17.5–20 min). DIA data was recorded as MSe (collision energy of 15–40 eV). Full scan spectra and their MS/MS spectra were acquired within a range of 50 to 1200 *m*/*z*. The UPLC-QTOF-MS data was acquired using Masslynx V4.1. and processed using Masslynx V4.2.

Additionally, some fractions were analyzed using high-resolution direct inlet mass spectrometry (DI-ESI MS (Q-TOF); HR DI-MS) on an Agilent 6546 QTOF in positive mode. The capillary voltage was set to 3000 V, gas temperature to 325 °C, gas flow rate to 10 L/min, and acquisition range to 50–1000 *m*/*z*.

### Plant Material

Shoots of *P. scouleri* and *P. torreyi* were collected from Isla de Todos Santos (31°48′25.63″N, 116°47′46.07″W, Ensenada, Baja California, Mexico), located within the Baja California Pacific Islands Biosphere Reserve. The specimens are cataloged in our herbarium under #10355 for *P. torreyi* and #10389 for *P. scouleri*. Additionally, the shoots were compared with previously identified shoots of *P torreyi*, and *P scouleri* kept at the marine macrophytes herbarium of the Faculty of Marine Sciences at the Autonomous University of Baja California. Plants were collected by scuba diving in a healthy and highly productive meadow (up to ∼4000 g DW m^–2^ of leaf biomass and ∼15000 shoots m^–2^) that extended from the intertidal to the shallow subtidal (max. Five m depth). The first sampling was done in February 2022 (winter), when *P. scouleri* shoots were collected from the bathymetric extremes of the meadow (intertidal versus 5 m depth). In the second sampling (May 2024), subtidal *P. torreyi* and *P. scouleri* shoots were collected at 3–5 m depth. In February 2022 the identification of *P. scouleri* was made by J.M. S.-G. based on the leaf morphology.^43^ In May 2024, the identification of each species was made based on the leaf morphology and inflorescence structures.^42^ The leaves of *P. torreyi* exhibited a rolled morphology with a rounded apex, in contrast to the flatter and wider leaves of *P. scouleri* with a slightly indented apex. For its part, the generative shoots of *P. torreyi* were longer than those of *P. scouleri*, reaching up to 45 cm and below 15 cm, respectively. They also produced multiple spathes, in contrast to the 1–2 spathes observed in *P. scouleri*. Additionally, the specimens were compared with the previously identified surfgrasses held at the marine macrophytes collection of the Faculty of Marine Sciences at the Autonomous University of Baja California. At each sampling time, ten samples (50–70 shoots each) were randomly collected from different patches separated at least 10 m apart. To avoid physiological stress associated with sun overexposure, subtidal plants were collected and kept in black mesh bags. Within 2 h, the collected plants were transported to the laboratory in coolers filled with seawater from the site. The leaves were then separated from the rhizomes and carefully cleaned of epiphytes and associated invertebrates. Any wounded or necrotic old leaves, almost fully covered by epiphytes, were discarded. The remaining leaves were briefly washed in distilled water to remove any adsorbed salts. They were then dried at room temperature until a constant weight was achieved.

### Extraction and Isolation

Plant material of *P. scouleri* (February 2022) (15 g) was used to extract and isolate phenolic compounds. The material was thoroughly mixed with 150 mL of a 50:50 (v/v) mixture of deionized water and methanol and then macerated at room temperature for 24 h. The extraction process was repeated three times, and the resulting extracts were pooled. The supernatant (15 mL) was reserved for HPLC analysis. The methanol was eliminated by evaporating the remaining solution under a vacuum. Subsequently, the aqueous solution was freeze-dried. The aqueous-methanolic extract was analyzed with HPLC-DAD, and the purification steps were monitored by both HPLC and NMR. The individual phenolics were isolated through repeated column chromatography on C18 reverse-phase silica gel, using gradient elution was done with H_2_O–MeOH as the mobile phase, starting from 95:5 to 50:50. HPLC and NMR also monitored the fractionation. The compounds were identified by analyzing their UV, NMR, and MS spectra and comparing them with published data.

#### 6-Hydroxyluteolin 6,3'Disulfate (**7**)

yellow amorphous powder; ^1^H and ^13^C NMR (850 and 212.5 MHz, DMSO-*d*_6_): see Table 2 and Figures S2–S5; HR-ESI-MS *m*/*z* 463.9640 [M + H]^+^; (calculated for C_15_H_11_O_13_S_2_, 463.9641; (error: −0.2 ppm)), *m*/*z* 383 [M-80 + H]^+^, *m*/*z* 303 [M-160 + H]^+^; UV Spectra (DAD online) 272 and 328 nm, Table 1.

#### 6-Hydroxyluteolin 7',4'-Sulfate (**10**)

yellow amorphous powder; ^1^H and ^13^C NMR (850 and 212.5 MHz, DMSO-*d*_6_): see Table 2 and Figures S2–S5; HR-ESI-MS *m*/*z* 463.9642 [M + H]^+^; (calculated for C_15_H_11_O_13_S_2_, 463.9641; (error: 0.2 ppm)), *m*/*z* 383 [M-80 + H]^+^, *m*/*z* 303 [M-160 + H]^+^; UV Spectra (DAD online) 280 and 330 nm, Table 1.

#### 6-Hydroxyluteolin 7,3'Disulfate (**12**)

yellow amorphous powder; ^1^H and ^13^C NMR (850 and 212.5 MHz, DMSO-*d*_6_): see Table 2 and Figures S2–S5; HR-ESI-MS *m*/*z* 463.9640 [M + H]^+^; (calculated for C_15_H_11_O_13_S_2_, 463.9641; (error: −0.2 ppm)), *m*/*z* 383 [M-80 + H]^+^, *m*/*z* 303 [M-160 + H]^+^; UV Spectra (DAD online) 282 and 332 nm, Table 1.

#### Luteolin 7,4'-Disulfate (**13**)

yellow amorphous powder; HR-ESI-MS *m*/*z* 447.9696 [M + H]^+^; (calculated for C_15_H_11_O_12_S_2_, 447.9692; (error: 0.9 ppm)); UV Spectra (DAD online) 282 and 335 nm, Table 1.

#### Luteolin 7,3'-Disulfate (**14**)

yellow amorphous powder; HR-ESI-MS *m*/*z* 447.9696 [M + H]^+^; (calculated for C_15_H_11_O_12_S_2_, 447.9692; (error: 0.9 ppm)); UV Spectra (DAD online) 270 and 320 nm, Table 1.

#### Nepetin 7,3'-Disulfate (**15**)

yellow amorphous powder; HR-ESI-MS *m*/*z* 477.9796 [M + H]^+^; (calculated for C_16_H_13_O_13_S_2_, 477.9798; (error: −0.4 ppm)), *m*/*z* 397 [M-80+H]^+^, *m*/*z* 317 [M-160+H]^+^; UV Spectra (DAD online) 274 and 332 nm, Table 1.

#### 6-Hydroxyluteolin 6-Sulfate (**16**)

yellow amorphous powder; ^1^H and ^13^C NMR (850 and 212.5 MHz, DMSO-*d*_6_): see Tables 4 and 5 and Figures S6–S10; HR-ESI-MS *m*/*z* 383.0081 [M + H]^+^; (calculated for C_15_H_11_O_10_S, 383.0083; (error: 2.1 ppm)), *m*/*z* 303 [M-80 + H]^+^; UV Spectra (DAD online) 274 and 338 nm, Table 1.

#### 6-Hydroxyluteolin 7-Sulfate (**17**)

yellow amorphous powder; ^1^H and ^13^C NMR (850 and 212.5 MHz, DMSO-*d*_6_): see Tables 4 and 5 and Figures S11–S13; HR-ESI-MS *m*/*z* 383.0081 [M + H]^+^; (calculated for C_15_H_11_O_10_S, 383.0083; (error: 2.1 ppm)), *m*/*z* 303 [M-80 + H]^+^; UV Spectra (DAD online) 282 and 346 nm, Table 1.

#### Scutellarien 6-Sulfate (**18**)

yellow amorphous powder; ^1^H and ^13^C NMR (850 and 212.5 MHz, DMSO-*d*_6_): see Tables 4 and 5 and Figures S14–S17; HR-ESI-MS *m*/*z* 367.0135 [M + H]^+^; (calculated for C_15_H_11_O_9_S, 367.0124; (error: 3 ppm)), *m*/*z* 287 [M-80 + H]^+^; UV Spectra (DAD online) 274 and 338 nm, Table 1.

#### Nodifloretin 6-Sulfate (**19**)

yellow amorphous powder; ^1^H and ^13^C NMR (850 and 212.5 MHz, DMSO-*d*_6_): see Tables 4 and 5 and Figures S18–S22; HR-ESI-MS *m*/*z* 397.0230 [M + H]^+^; (calculated for C_16_H_13_O_10_S, 397.0229; (error: 0.3 ppm), *m*/*z* 317 [M-80 + H]^+^; UV Spectra (DAD online) 282 and 334 nm, Table 1.

#### Nepetin 7-Sulfate (**20**)

yellow amorphous powder. LR-ESI-MS *m*/*z* 397 [M + H]^+^; *m*/*z* 317 [M-80 + H]^+^; UV Spectra (DAD online) 282 and 344 nm, Table 1.

#### Nodifloretin 7-Sulfate (**22**)

yellow amorphous powder; ^1^H and ^13^C NMR (850 and 212.5 MHz, DMSO-*d*_6_): see Tables 4 and 5 and Figures S23–S26; HR-ESI-MS *m*/*z* 397.0230 [M + H]^+^; (calculated for C_16_H_13_O_10_S, 397.0229; (error: 0.3 ppm), *m*/*z* 317 [M-80 + H]^+^; UV Spectra (DAD online) 282 and 344 nm, Table 1.

#### Luteolin 4'-Sulfate (**24**)

yellow amorphous powder; ^1^H and ^13^C NMR (850 and 212.5 MHz, DMSO-*d*_6_): see Tables 4 and 5 and Figures S27–S30; HR-ESI-MS *m*/*z* 287.0550 [M-80+H]^+^; UV Spectra (DAD online) 268 and 332 nm, Table 1.

#### Luteolin 3'-Sulfate (**25**)

yellow amorphous powder, ^1^H NMR (850 MHz, DMSO-*d*_6_): see Table 4; HR-ESI-MS *m*/*z* 367.0135 [M + H]^+^; (calculated for C_15_H_11_O_9_S, 367.0124; (error: 3 ppm)), *m*/*z* 287 [M-80 + H]^+^; UV Spectra (DAD online) 268 and 336 nm, Table 1.

#### Hispidulin 7-Sulfate (**26**)

yellow amorphous powder. HR-ESI-MS *m*/*z* 381.0291 [M + H]^+^; (calculated for C_16_H_13_O_9_S, 381.0280; (error: 2.9 ppm)), *m*/*z* 301 [M-80 + H]^+^; UV Spectra (DAD online) 272 and 336 nm, Table 1.

#### Jaceosidin 7-Sulfate (**28**)

yellow amorphous powder; ^1^H and ^13^C NMR (850 and 212.5 MHz, DMSO-*d*_6_): see Tables 4 and 5 and Figures S31–S33; HR-ESI-MS *m*/z 411.0381[M + H]^+^; (calculated for C_17_H_15_O_10_S, 411.0386; (error: −1.2 ppm)); UV Spectra (DAD online) 272 and 346 nm, Table 1.

### Quantitative Determination

Plant materials of *P. scouleri* and *P. torreyi* collected in May 2024 were used for qualitative and quantitative comparative analysis. The materials were cut into small pieces and extracted with 50% aqueous methanol, the phenolic content of the extracts characterized by HPLC-DAD and LR/HR–LCMS. Two g of dried plant material was weighed and extracted with 50 mL of 50% aqueous methanol for 2 h at room temperature. This process was repeated two additional times. Three replicate samples were made for each plant. Prior to injection, the solutions were filtered through a 0.45 μm Millipore membrane filter. HPLC calibration curves of Luteolin (≥90% (HPLC), Sigma-Aldrich, Sigma-Aldric, St. Louis, MO, USA) and rosmarinic acid (≥98% (HPLC), Sigma-Aldrich) were used to determine the quantitative amounts of flavonoids and phenolic acids, respectively. The results are presented as milligrams luteolin or rosmarinic acid equivalents ± one standard deviation (SD) per gram of dry weight (DW) plant material. Two sample *t* tests assuming unequal variances with a *p*-value of 0.05 were used to determine if the means of two different measurements were equal or not. Standard error bars were calculated using the STDEV. The *p* function in Excel represents one standard deviation (n = 3 or some replicates).

## Supporting Information Available

HPLC profiles of crude extracts of *P. scouleri* collected in February 2022 and May 2024 (recorded at 330 ± 20 nm) (Figure S1); 1D and 2D NMR Spectra of compounds **7**, **10**, **12**, **16**-**19**, **22**, **24** and **28** (Figures S2–S32) (PDF)
