# Supplementary material for: Sulfated Flavonoids from Phyllospadix (Zosteraceae) Taxa from Baja California, Mexico
Source: ACS Omega. 2025 Feb 7;10(6):6079–91. doi: 10.1021/acsomega.4c10402 (PMC11840769; doi:10.1021/acsomega.4c10402)
Supplement: Supplementary file 1 — ao4c10402_si_001.pdf [file ao4c10402_si_001.pdf]

SUPPLEMENTARY MATERIAL

Sulfated flavonoids from Phyllospadix (Zosteraceae) taxa from Baja California, Mexico

*Diego Rodríguez-Hernández[1], Jose Miguel Sandoval-Gil[2], Kjersti Hasle Enerstvedt[1], Susana Villa Gonzalez[2], Alejandra Ferreira-Arrieta[3], Alexandros G. Asimakopoulos[3], and Monica Jordheim[1] **

[1]Department of Chemistry, University of Bergen, Allegaten 41, NO-5007 Bergen, Norway.

[2]Universidad Autónoma de Baja California, Instituto de Investigaciones Oceanólogicas, 22830, Ensenada, Baja California, Mexico.

[3]Department of Chemistry, Norwegian University of Science and Technology (NTNU) Trondheim, 7491, Norway.

Table of contents

Figure S1: HPLC profiles of crude extracts of *P, scouleri*…………….……………S2

Figure S2-S33: 1D and 2D NMR Spectra…………………………………………S3-S18

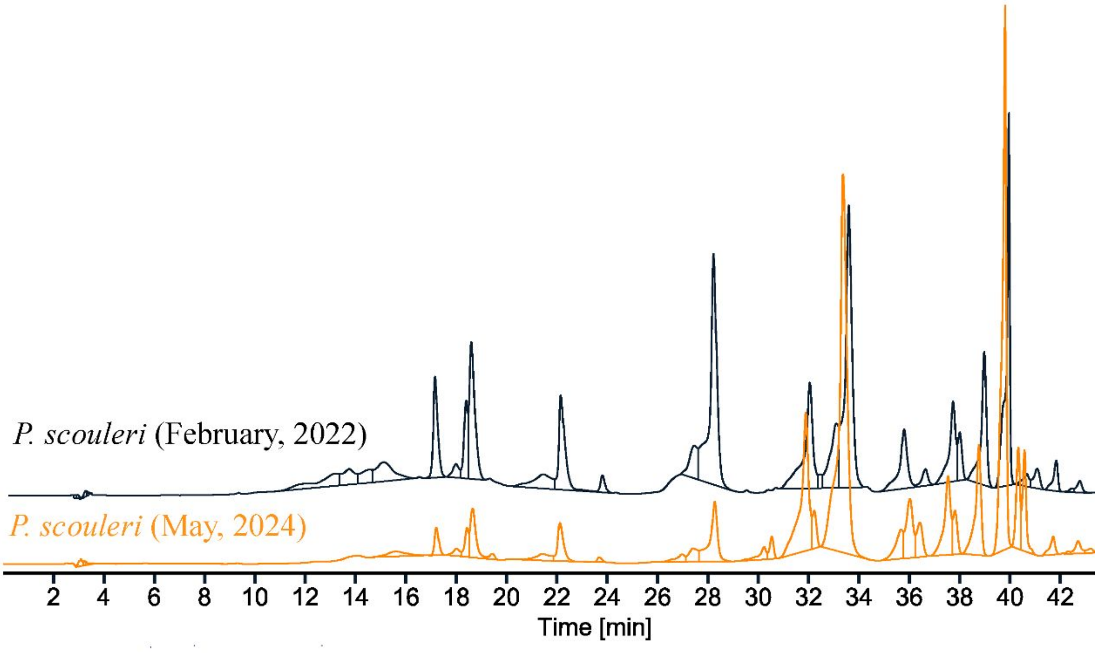

Figure S1: HPLC profiles of crude extracts of *P. scouleri* collected in February 2022 and May 2024 (recorded at 330±20 nm).

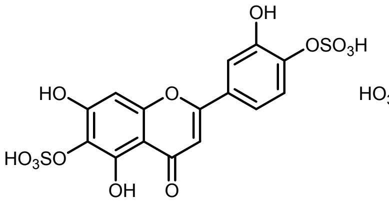

7

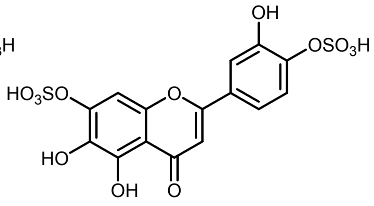

10

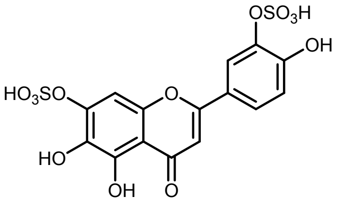

12

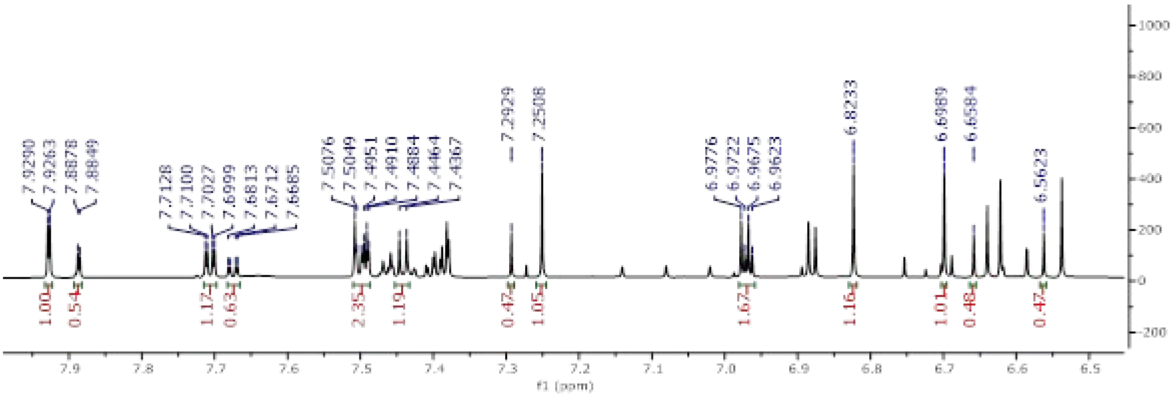

Figure S2: ^{1}H NMR spectrum of **7, 10**, **12** (850 MHz, DMSO-d_6).

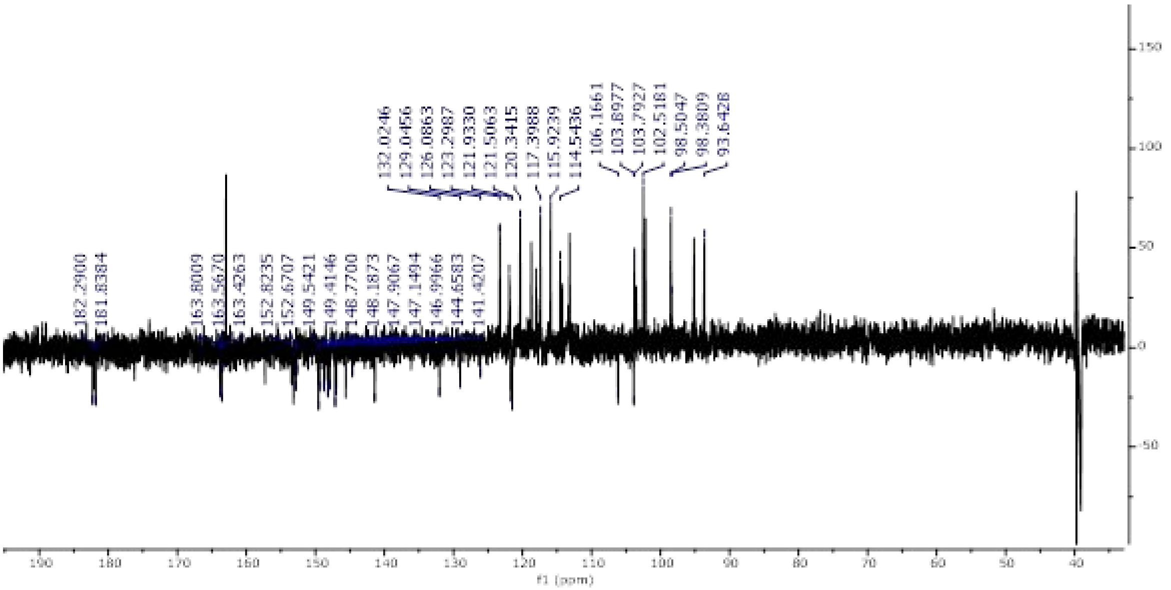

Figure S3: ^{13}C NMR spectrum of **7, 10**, **12** (212.5 MHz, DMSO-d_6).

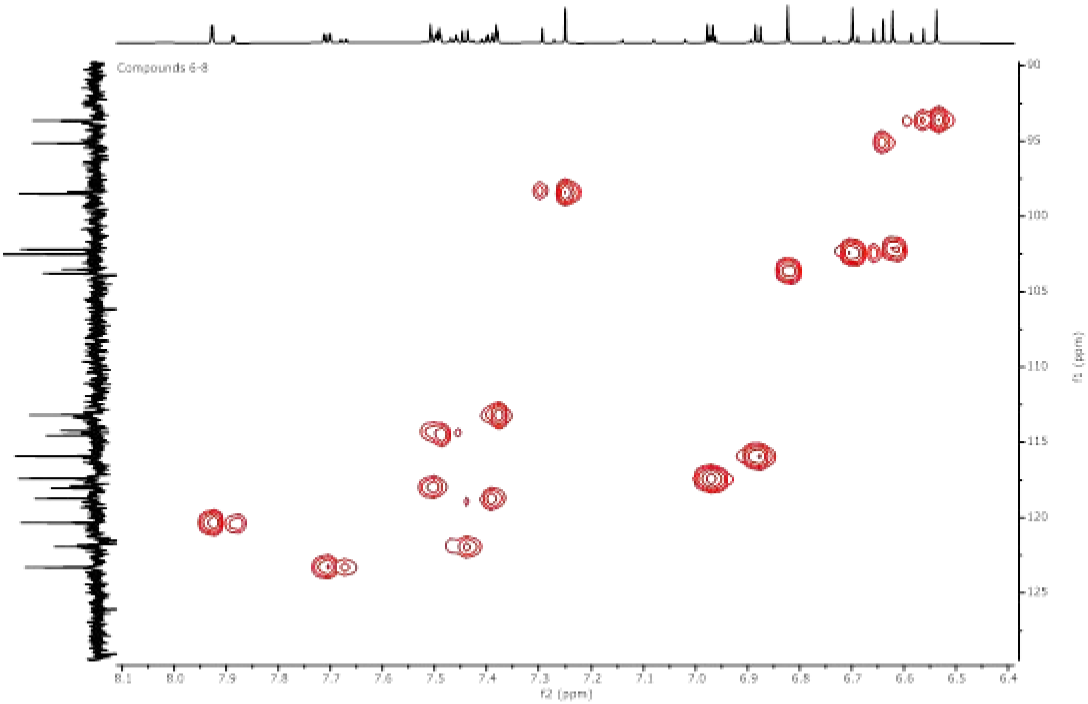

Figure S4: HSQC spectrum of **7**, **10**, **12** (DMSO-d_6).

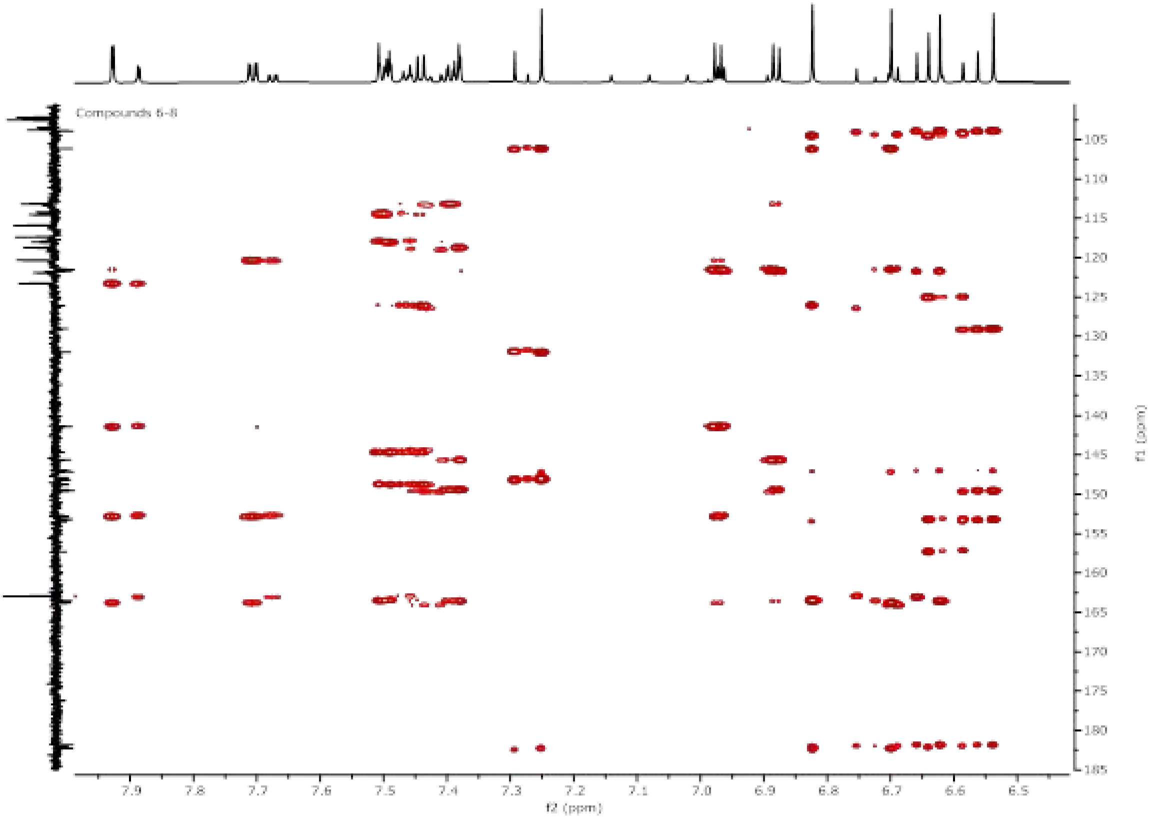

Figure S5: HMBC spectrum of **7**, **10**, **12** (DMSO-d_6).

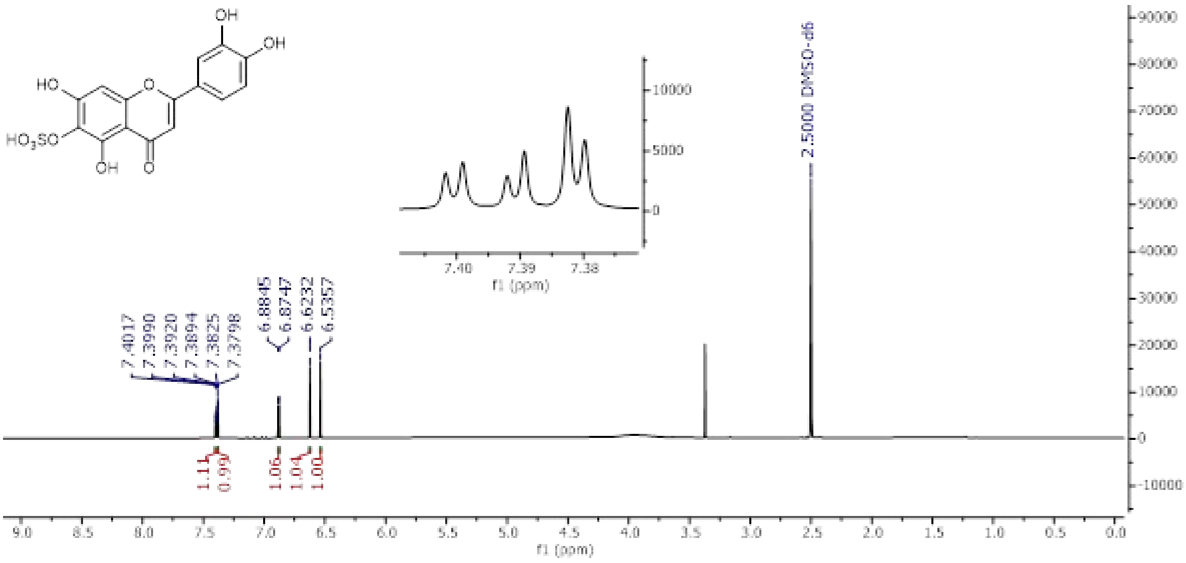

Figure S6: ^{1}H NMR spectrum of **7** (850 MHz, DMSO-d_6).

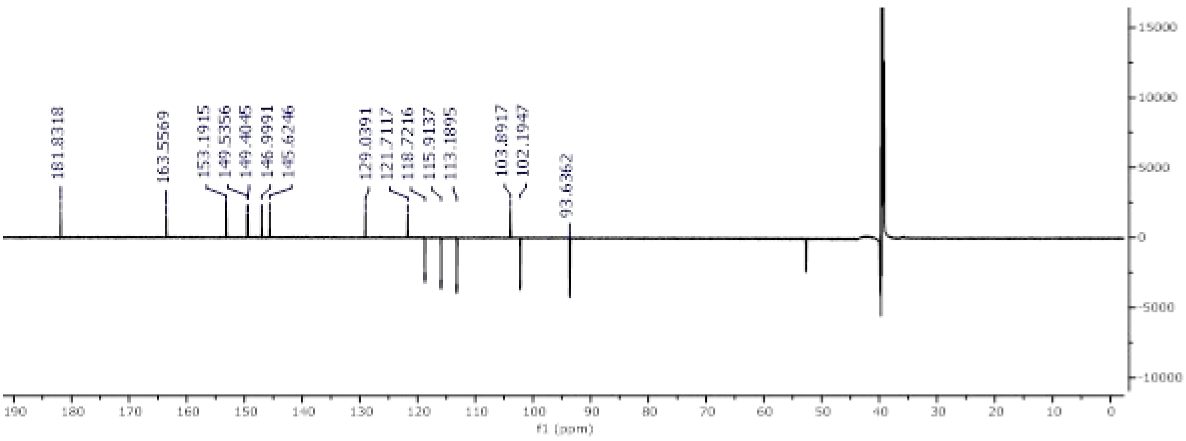

Figure S7: ^{13}C NMR spectrum of **16** (212.5 MHz, DMSO-d_6).

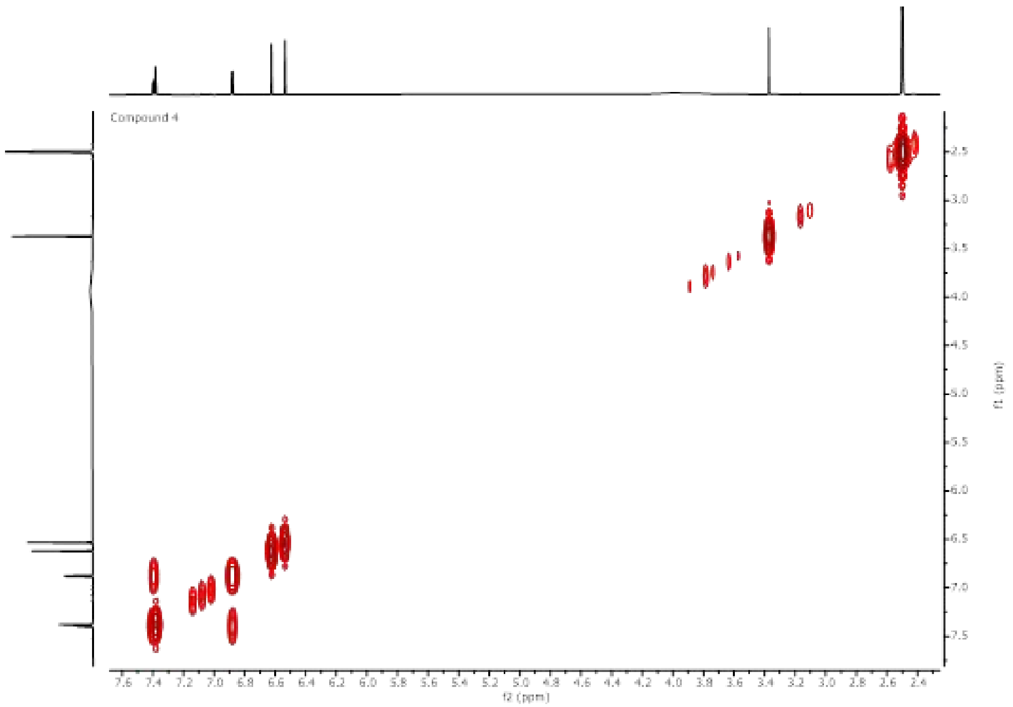

Figure S8: COSY spectrum of **16** (DMSO-d_6).

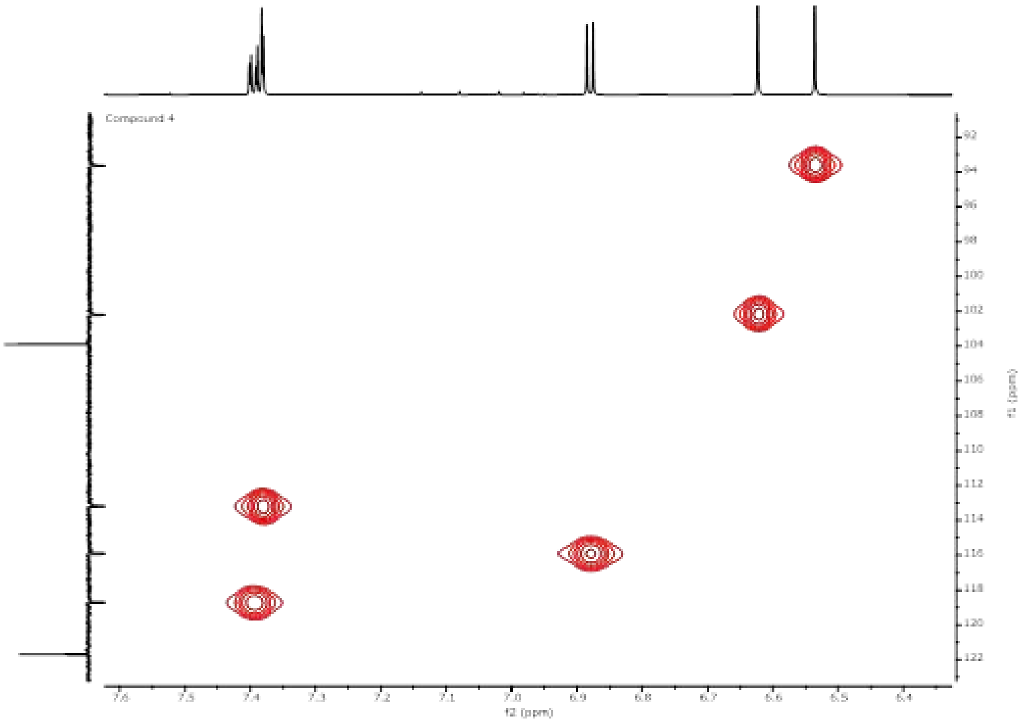

Figure S9: HSQC spectrum of **16** (DMSO-d_6).

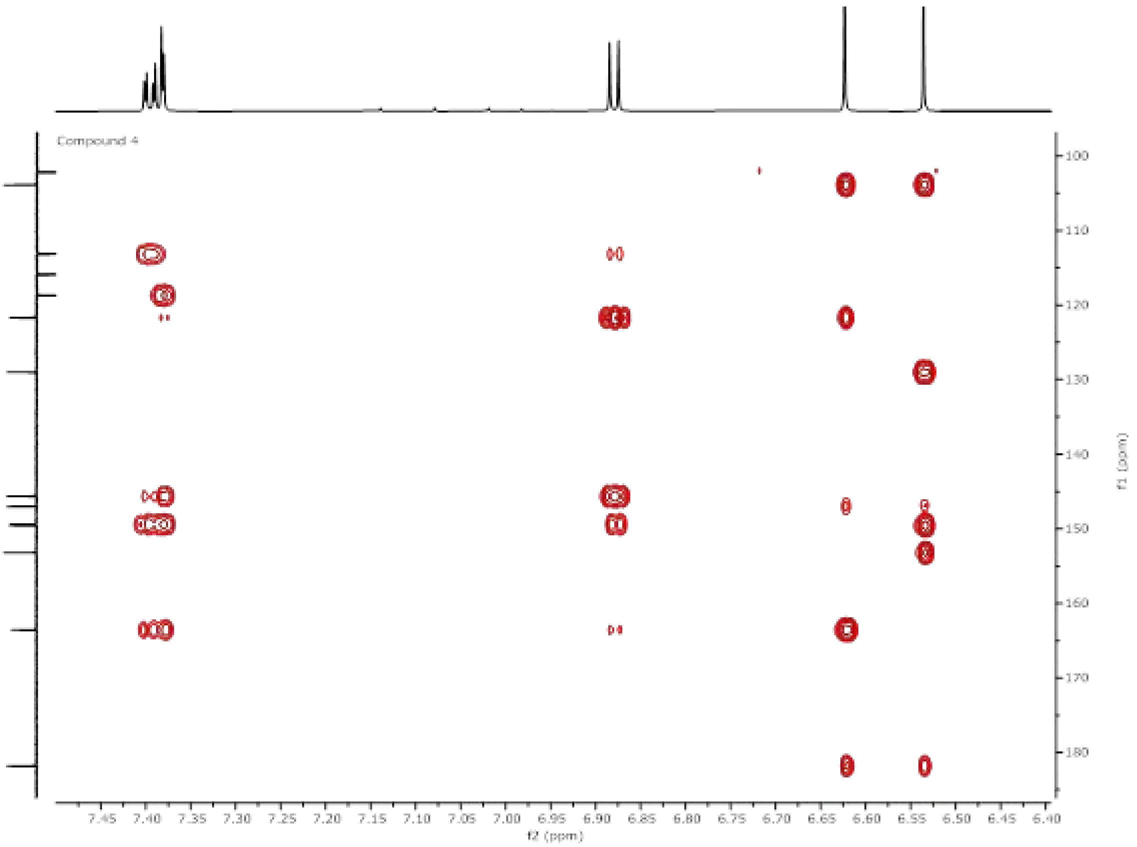

Figure S10: HMBC spectrum of **16** (DMSO-d_6)

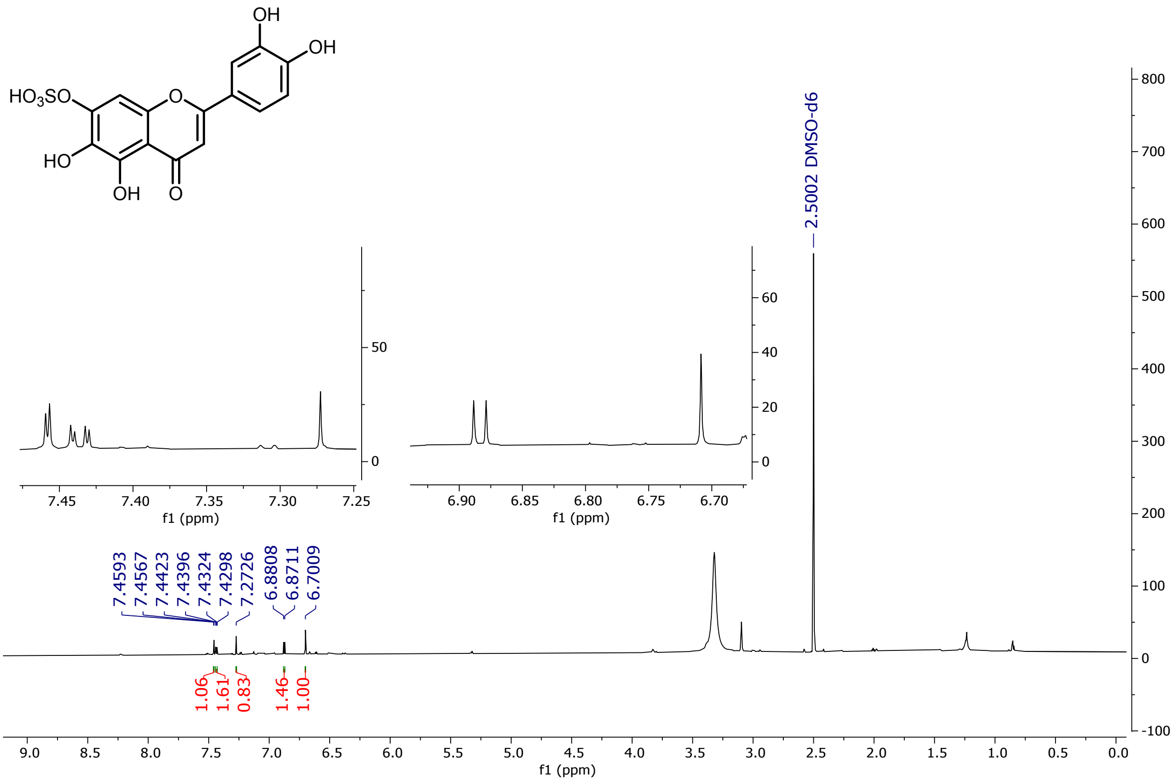

Figure S11: ^{1}H NMR spectrum of **17** (850 MHz, DMSO-d_6).

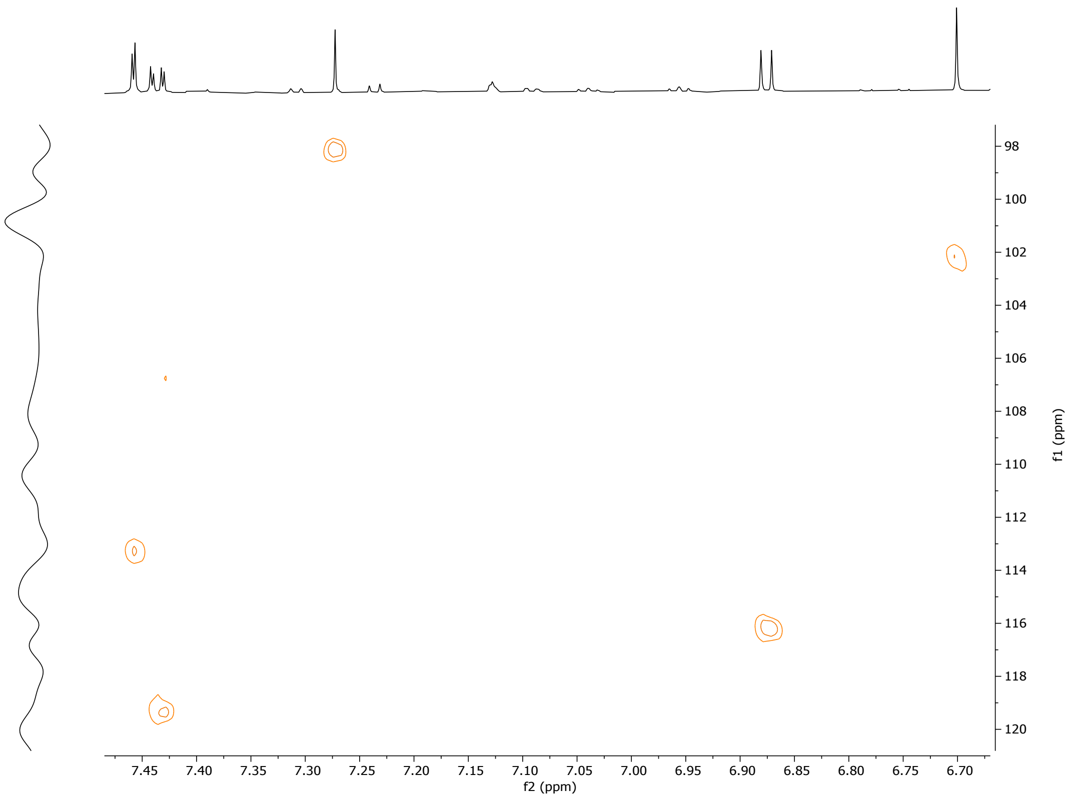

Figure S12: HSQC spectrum of **17** (DMSO-d_6).

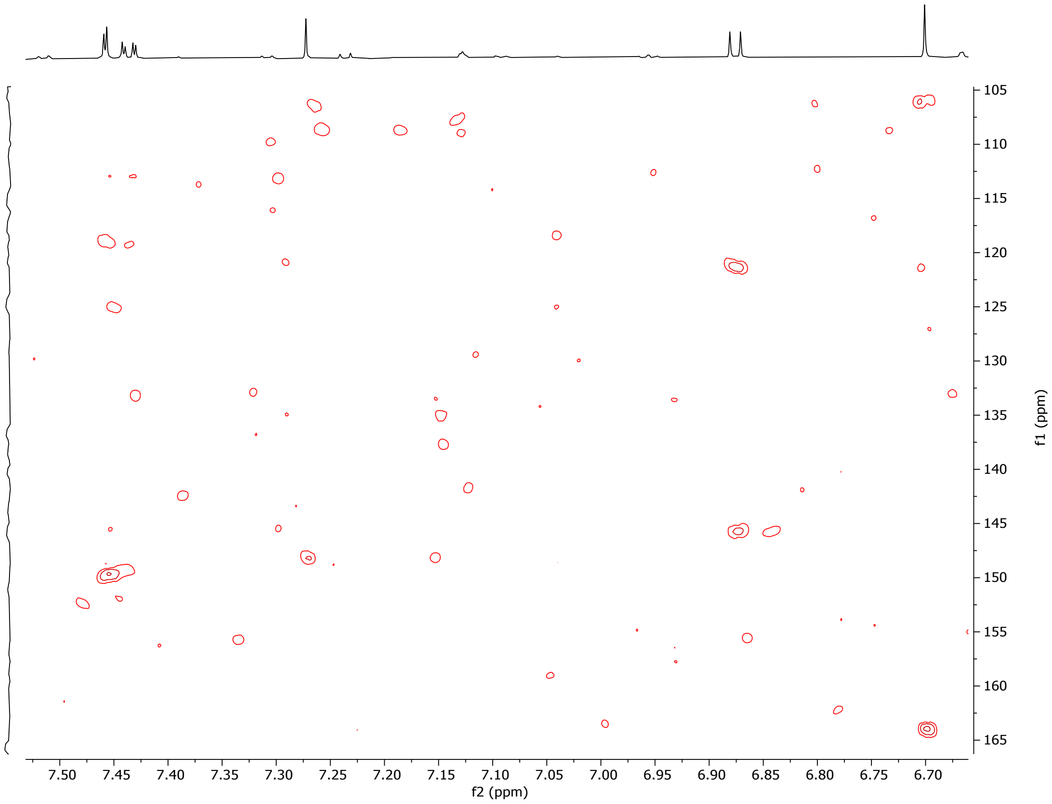

Figure S13: HMBC spectrum of **17** (DMSO-d_6).

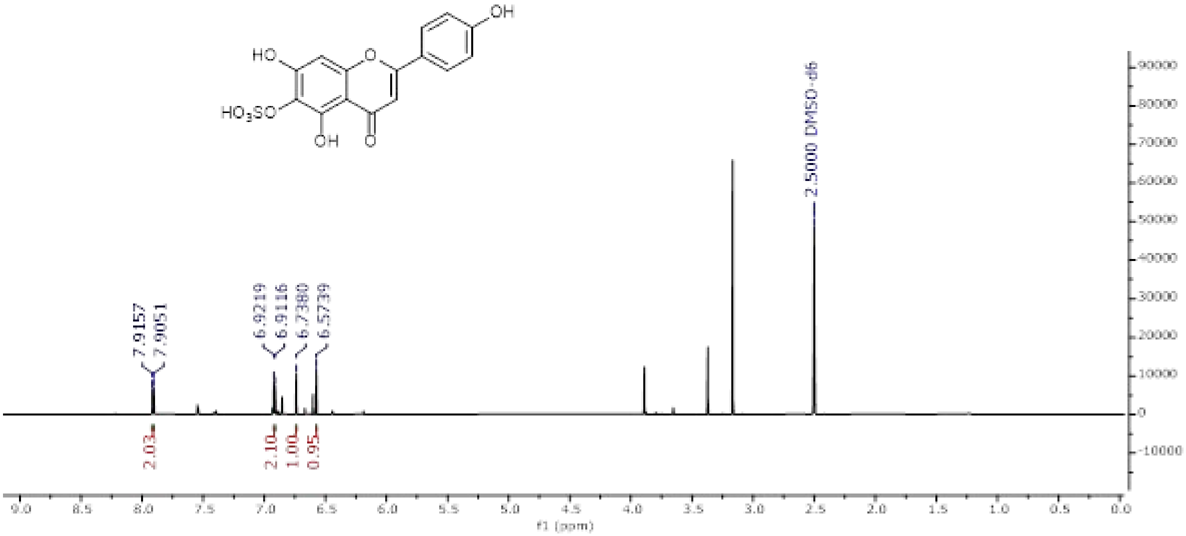

Figure S14: ^{1}H NMR spectrum of **18** (850 MHz, DMSO-d_6).

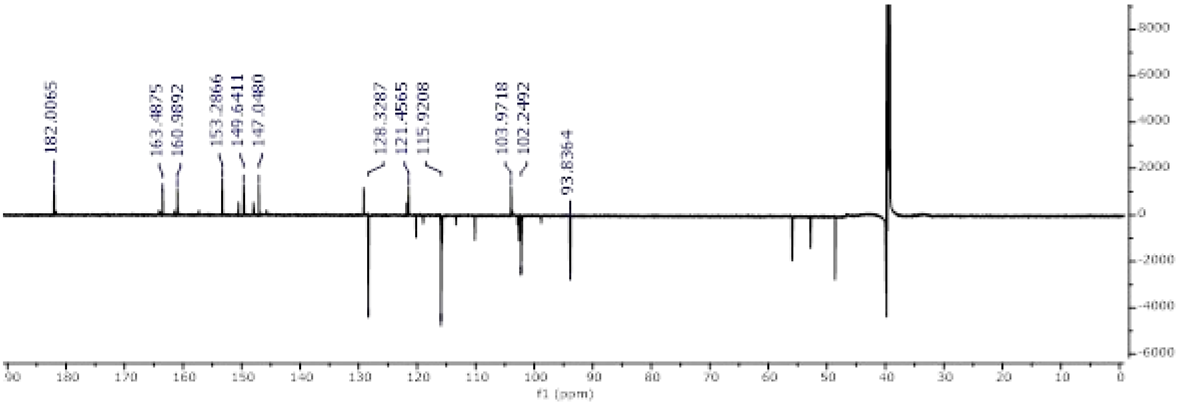

Figure S15: ^{13}C NMR spectrum of **18** (212.5 MHz, DMSO-d_6).

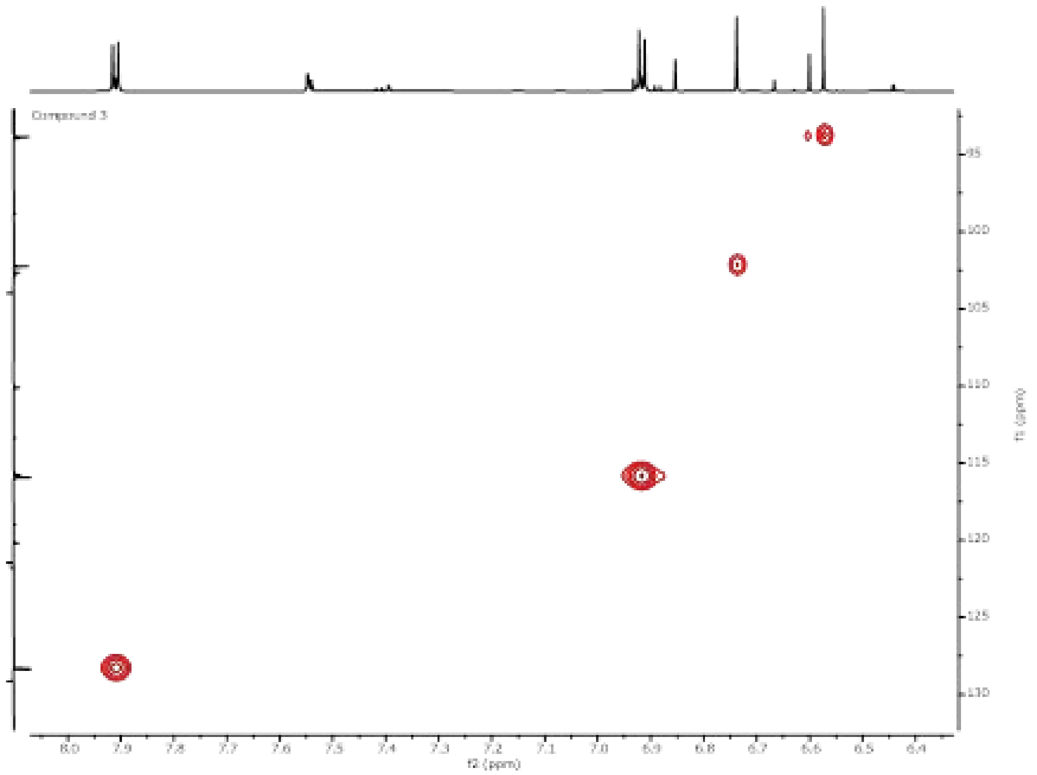

Figure S16: HSQC spectrum of **18** (DMSO-d_6).

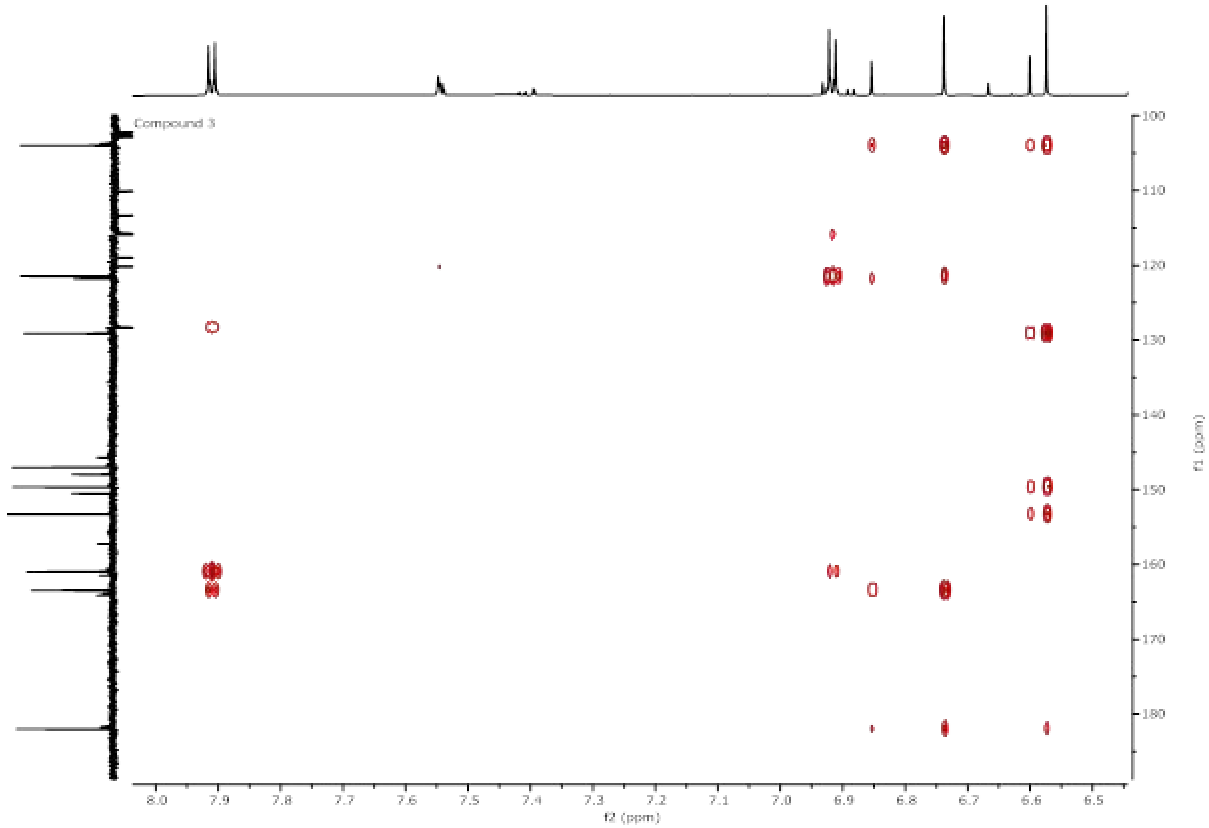

Figure S17: HMBC spectrum of **18** (DMSO-d_6).

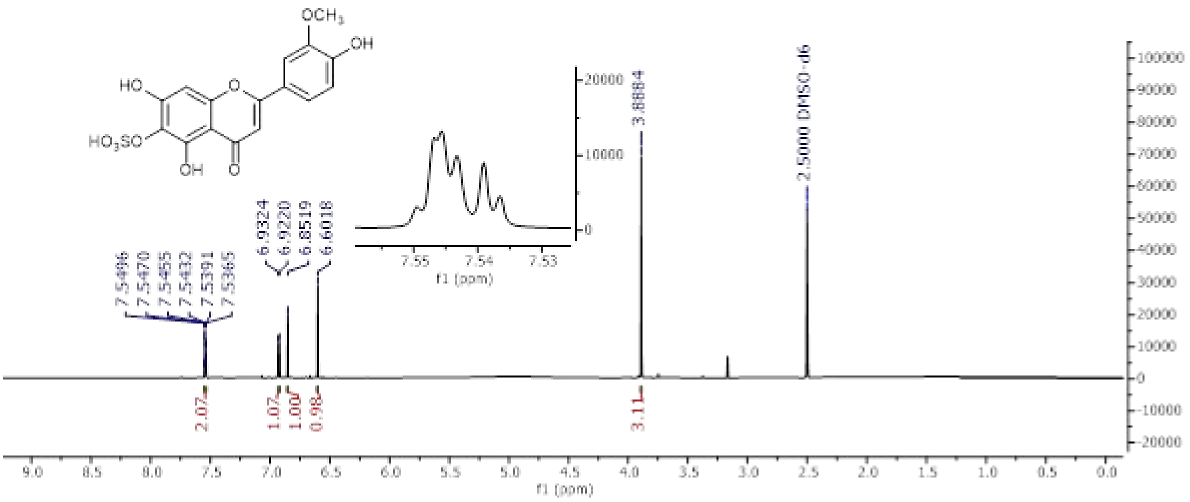

Figure S18: ^{1}H NMR spectrum of **19** (850 MHz, DMSO-d_6).

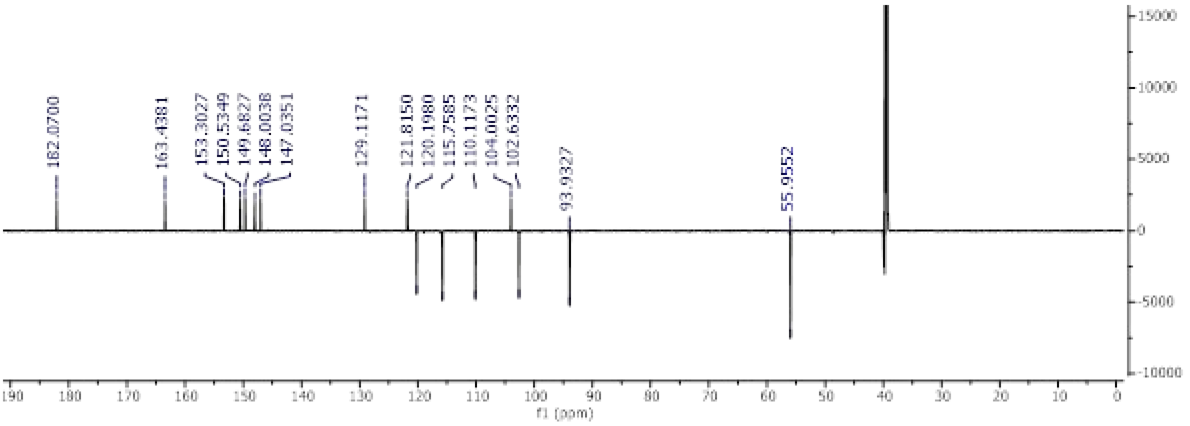

Figure S19: ^{13}C NMR spectrum of **19** (212.5 MHz, DMSO-d_6).

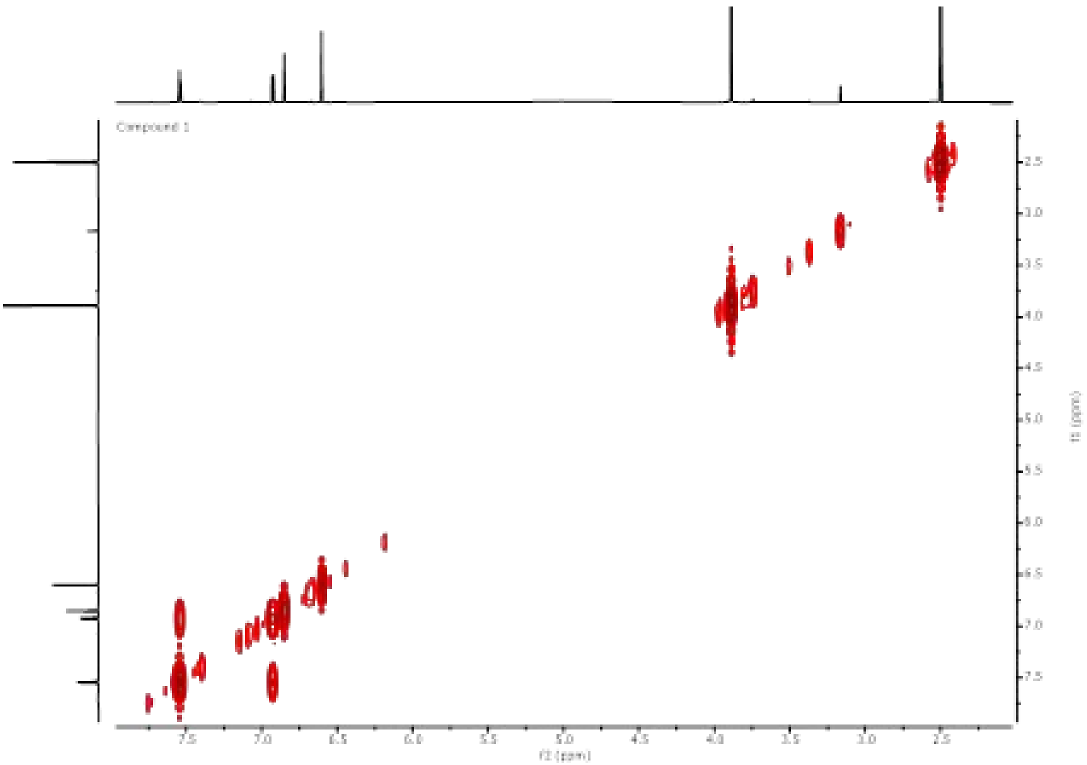

Figure S20: COSY spectrum of **19** (DMSO-d_6).

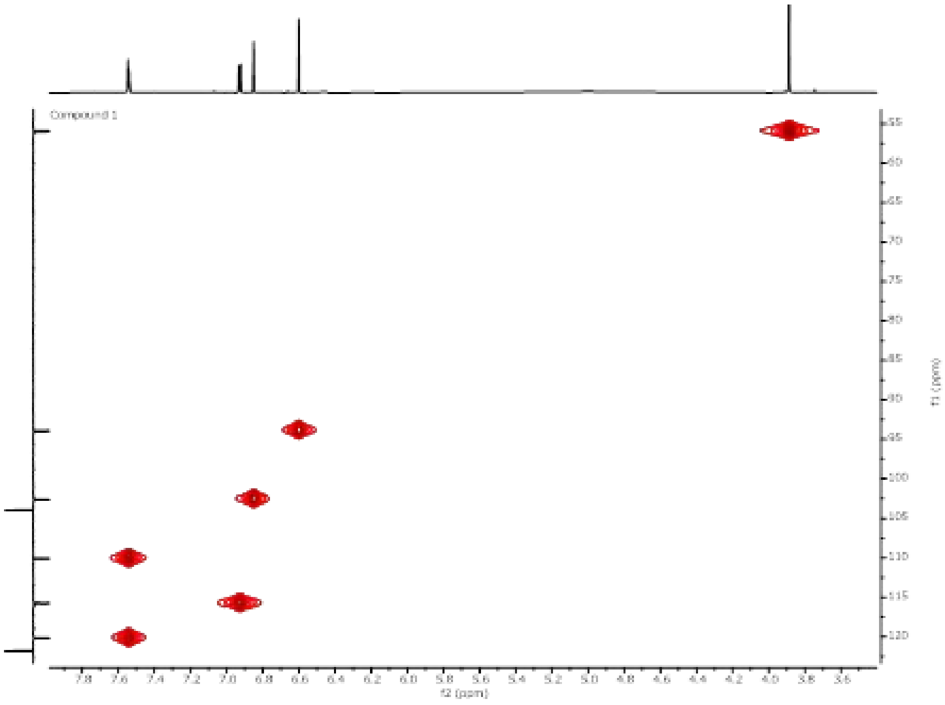

Figure S21: HSQC spectrum of **19** (DMSO-d_6).

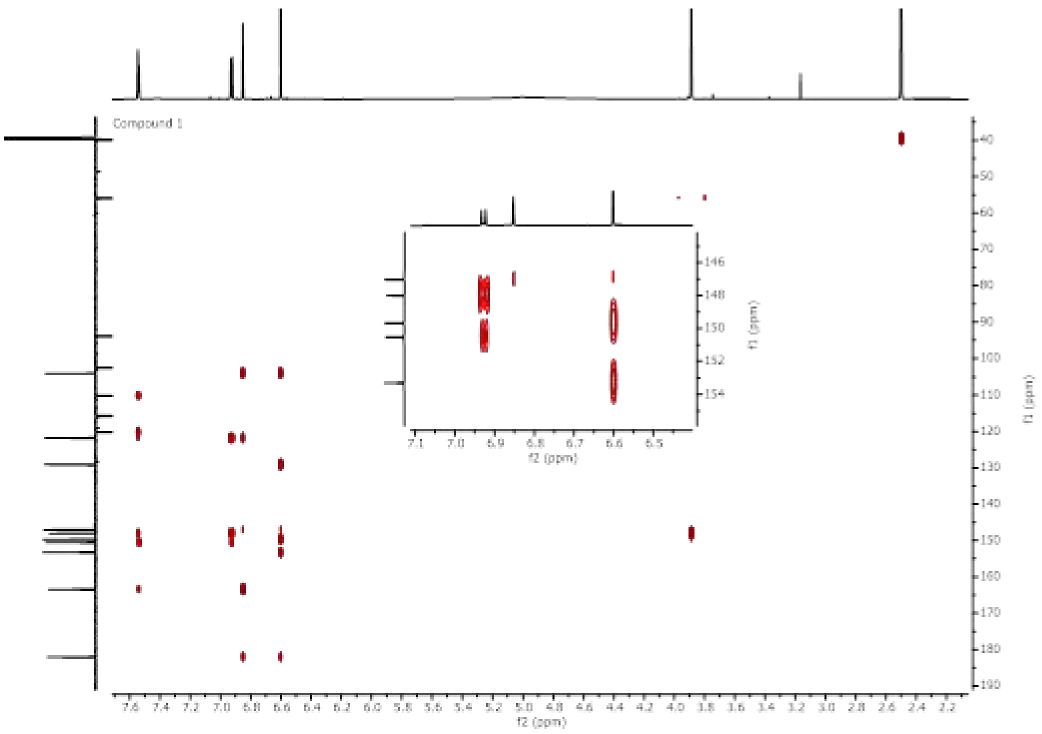

Figure S22: HMBC spectrum of **19** (DMSO-d_6).

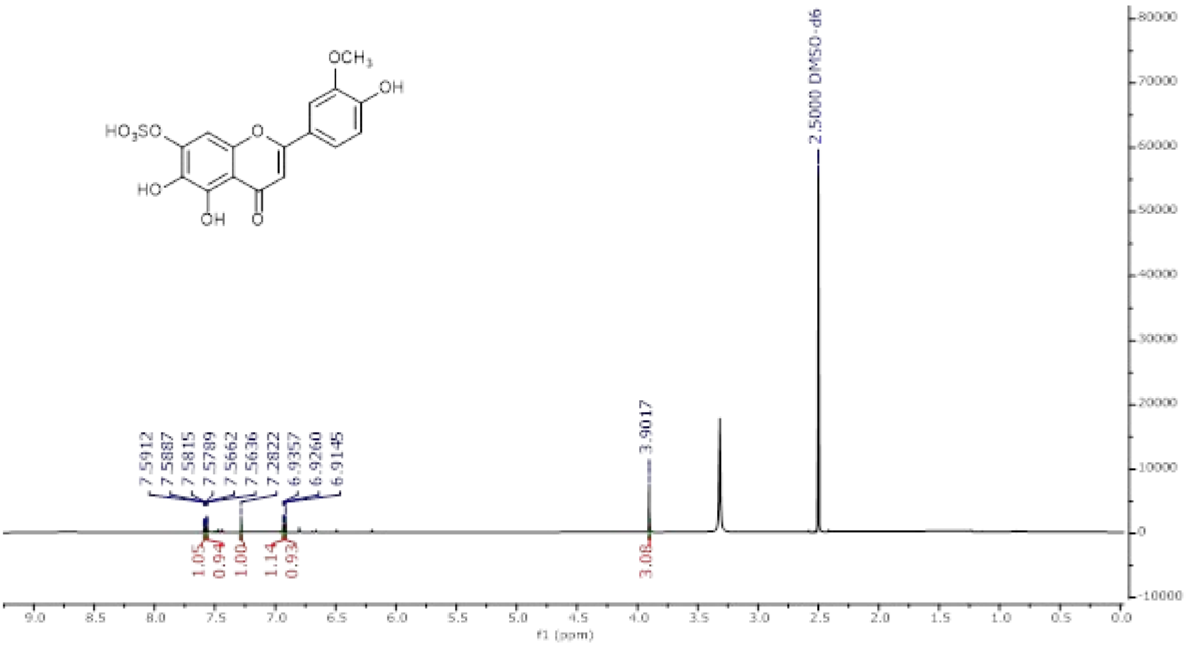

Figure S23: ^1H NMR spectrum of **22** (850 MHz, DMSO-d_6).

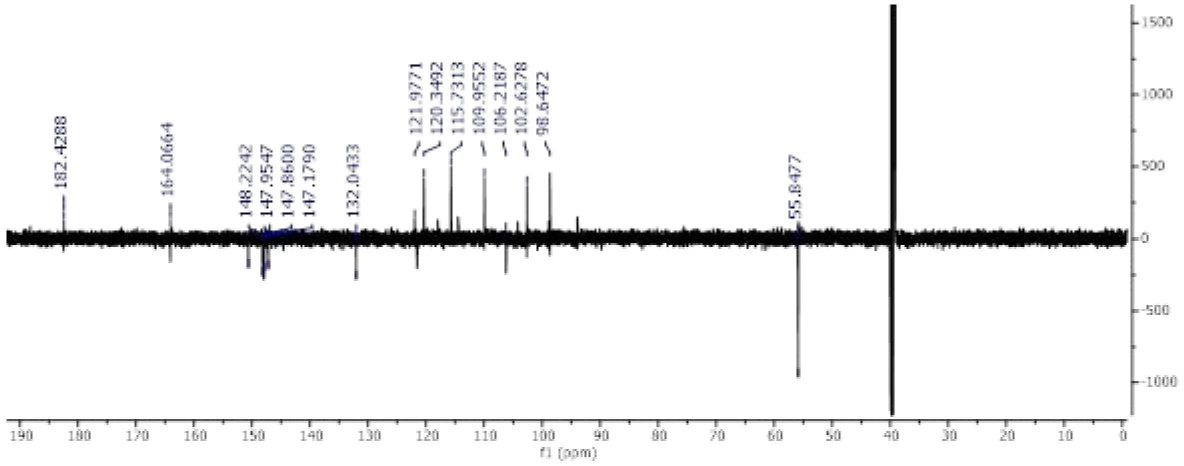

Figure S24: ^{13}C NMR spectrum of **22** (212.5 MHz, DMSO-d_6).

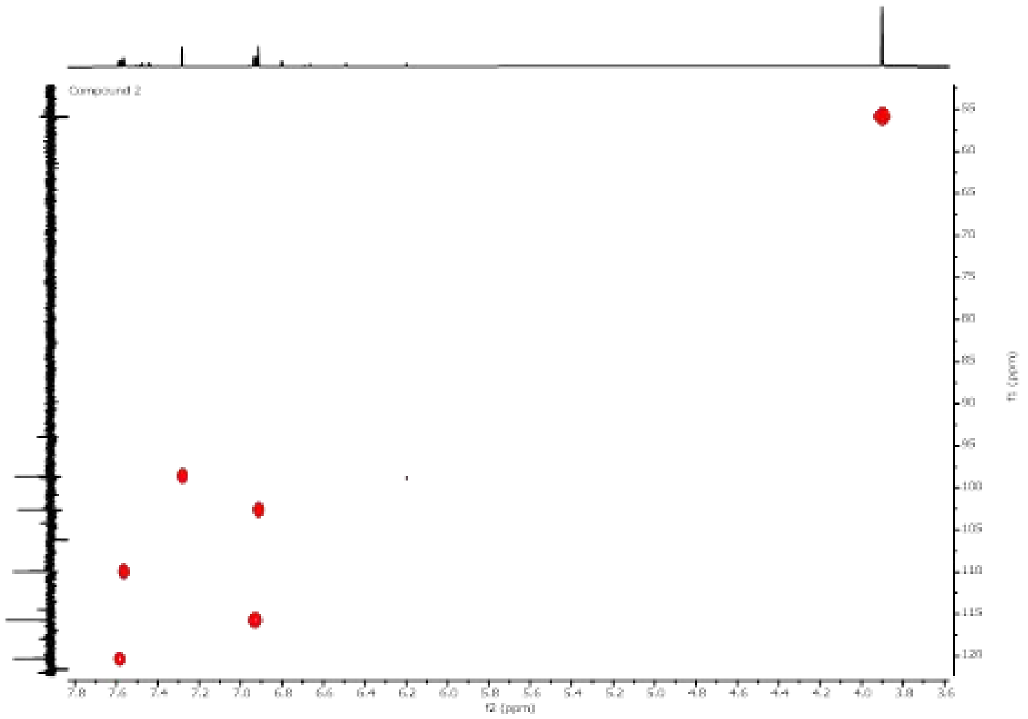

Figure S25: HSQC spectrum of **22** (DMSO-d_6).

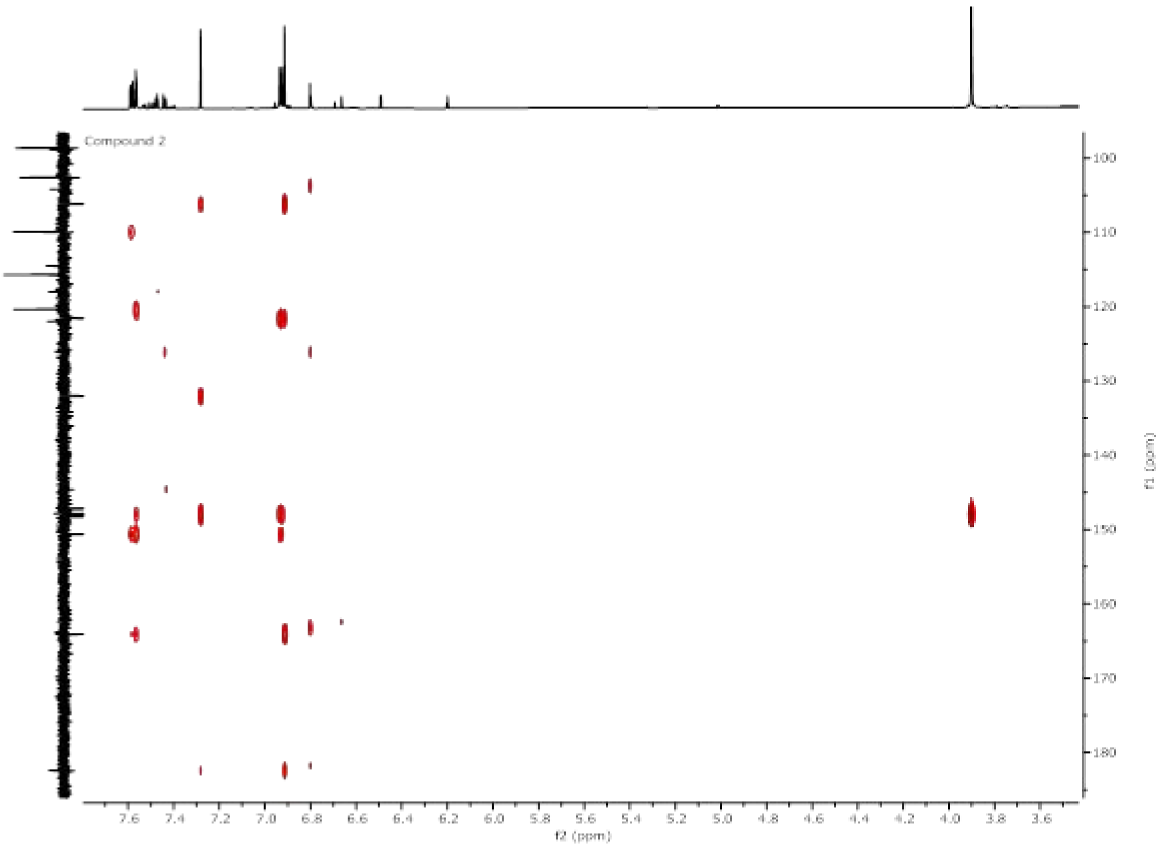

Figure S26: HMBC spectrum of **22** (DMSO-d_6).

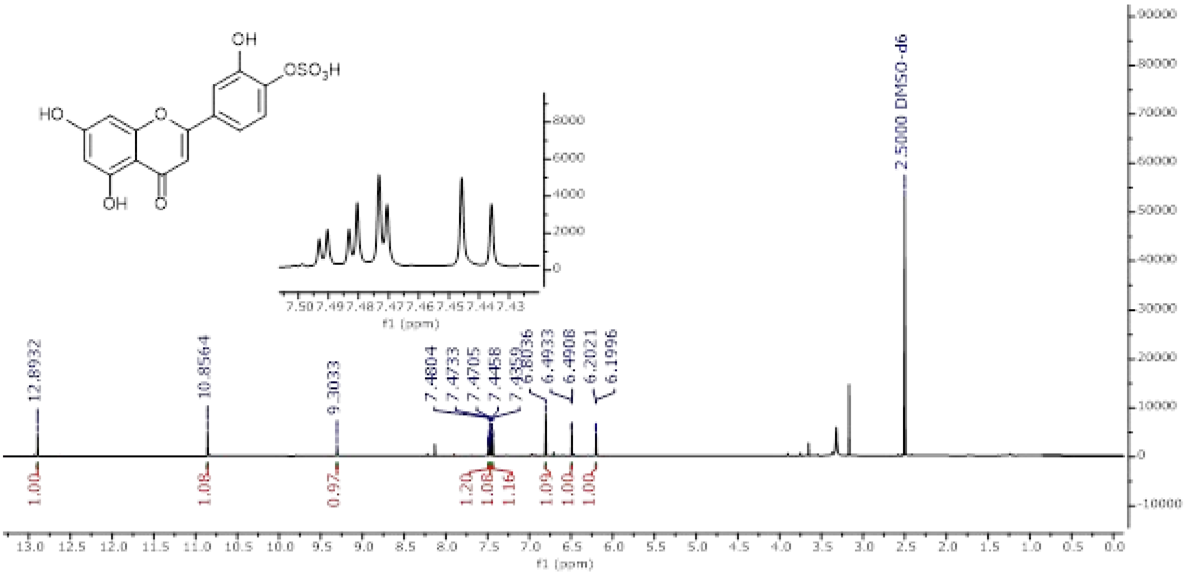

Figure S27: ^{1}H NMR spectrum of **24** (850 MHz, DMSO-d_6).

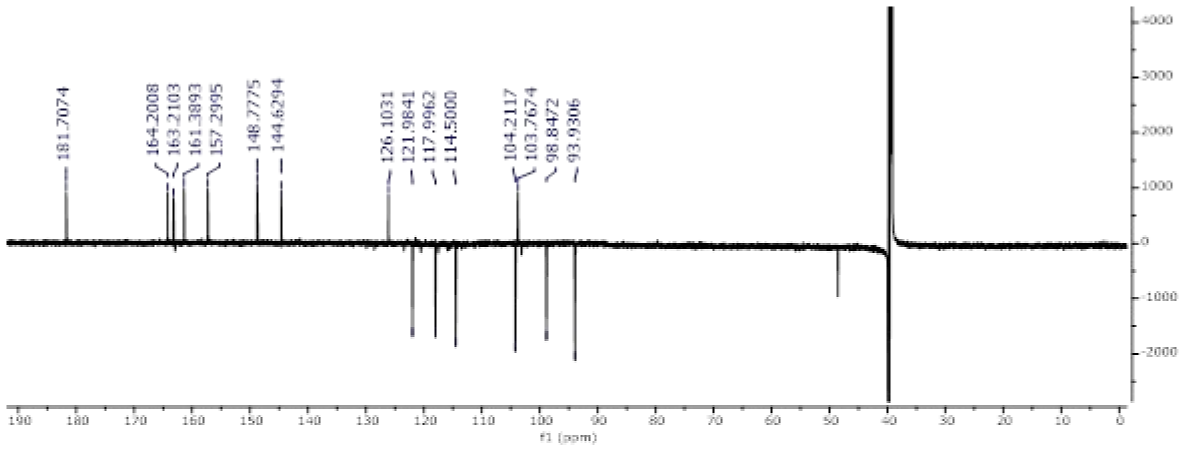

Figure S28: ^{13}C NMR spectrum of **24** (212.5 MHz, DMSO-d_6).

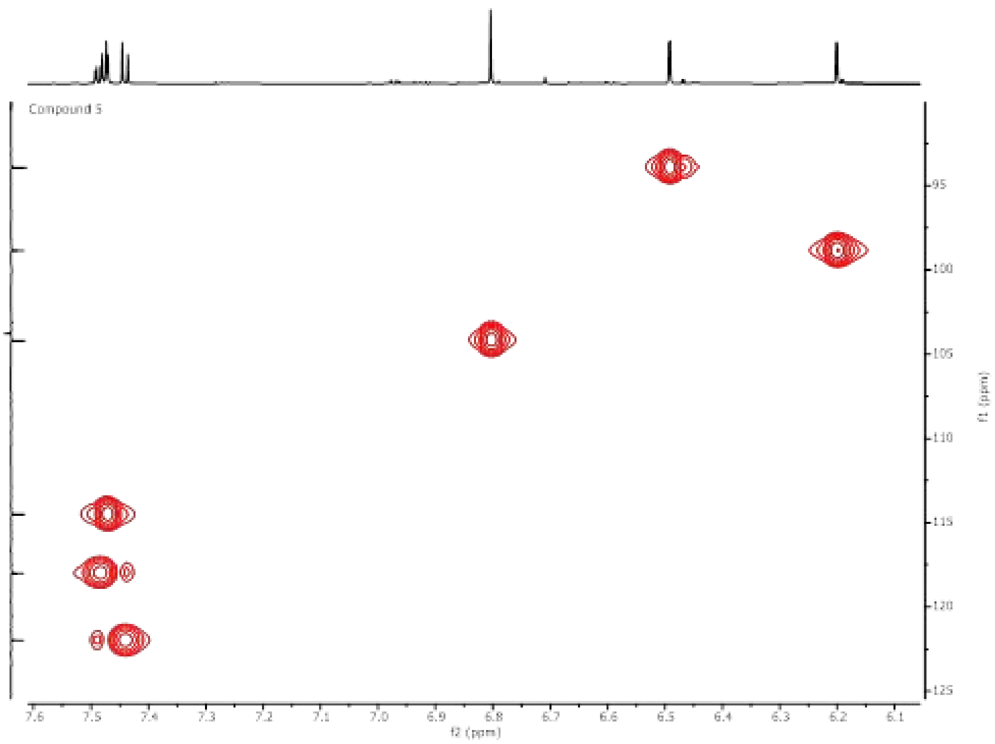

Figure S29: HSQC spectrum of **24** (DMSO-d_6).

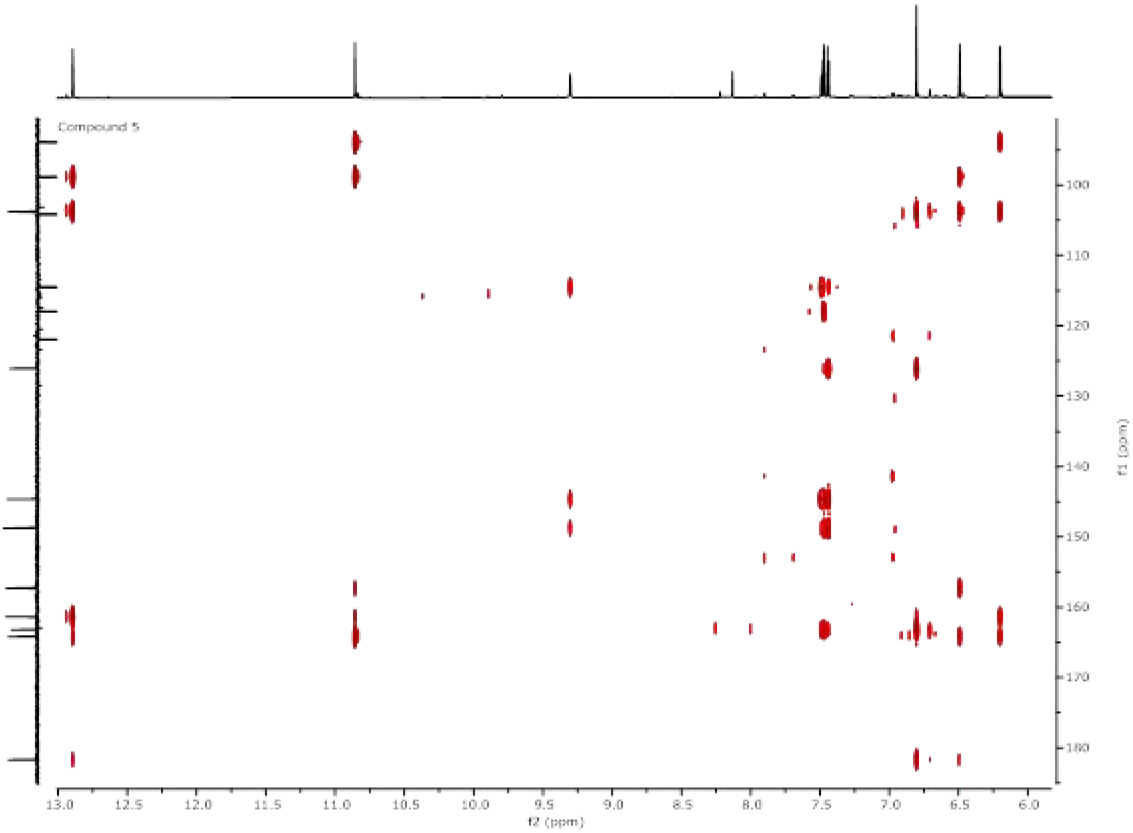

Figure S30: HMBC spectrum of **24** (DMSO-d_6).

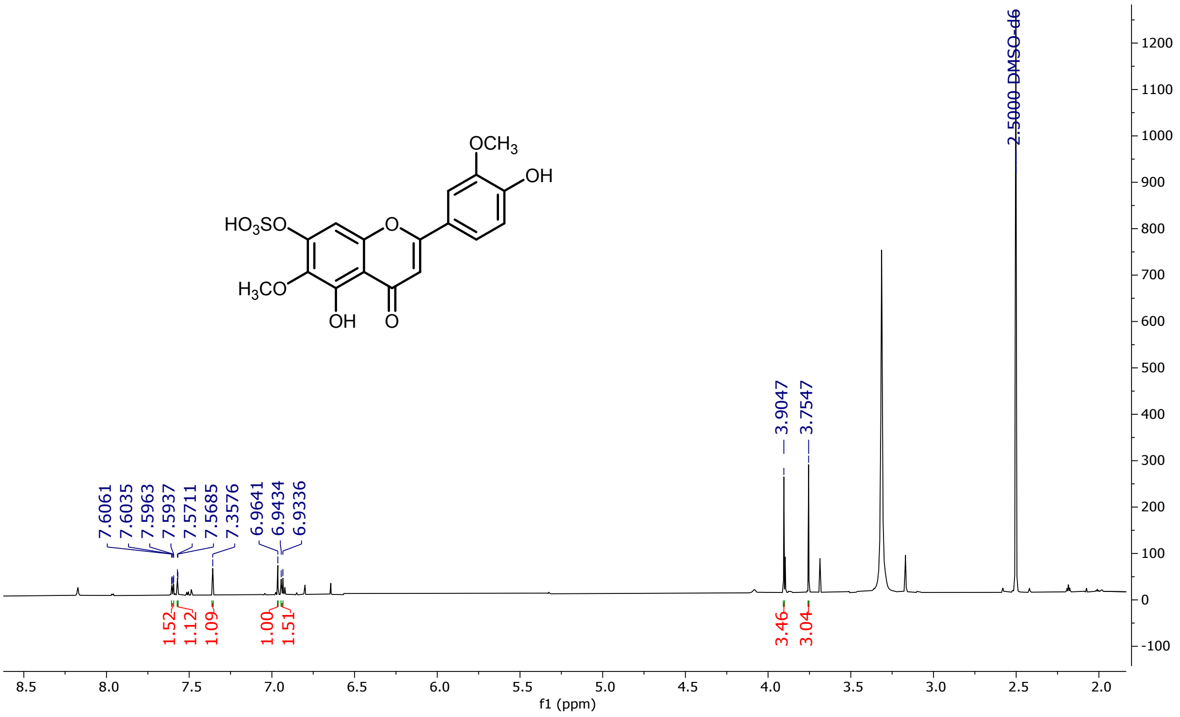

Figure S31: ^{1}H NMR spectrum of **28** (850 MHz, DMSO-d_6).

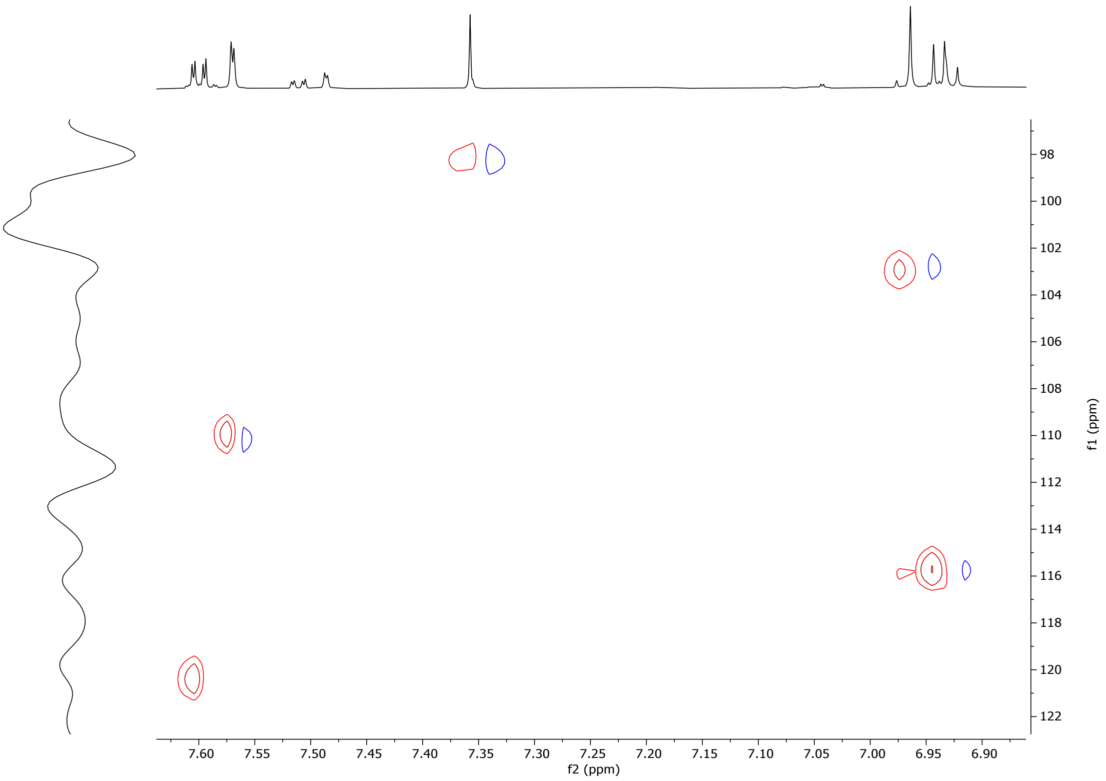

Figure S32: HSQC spectrum of **28** (DMSO-d_6).

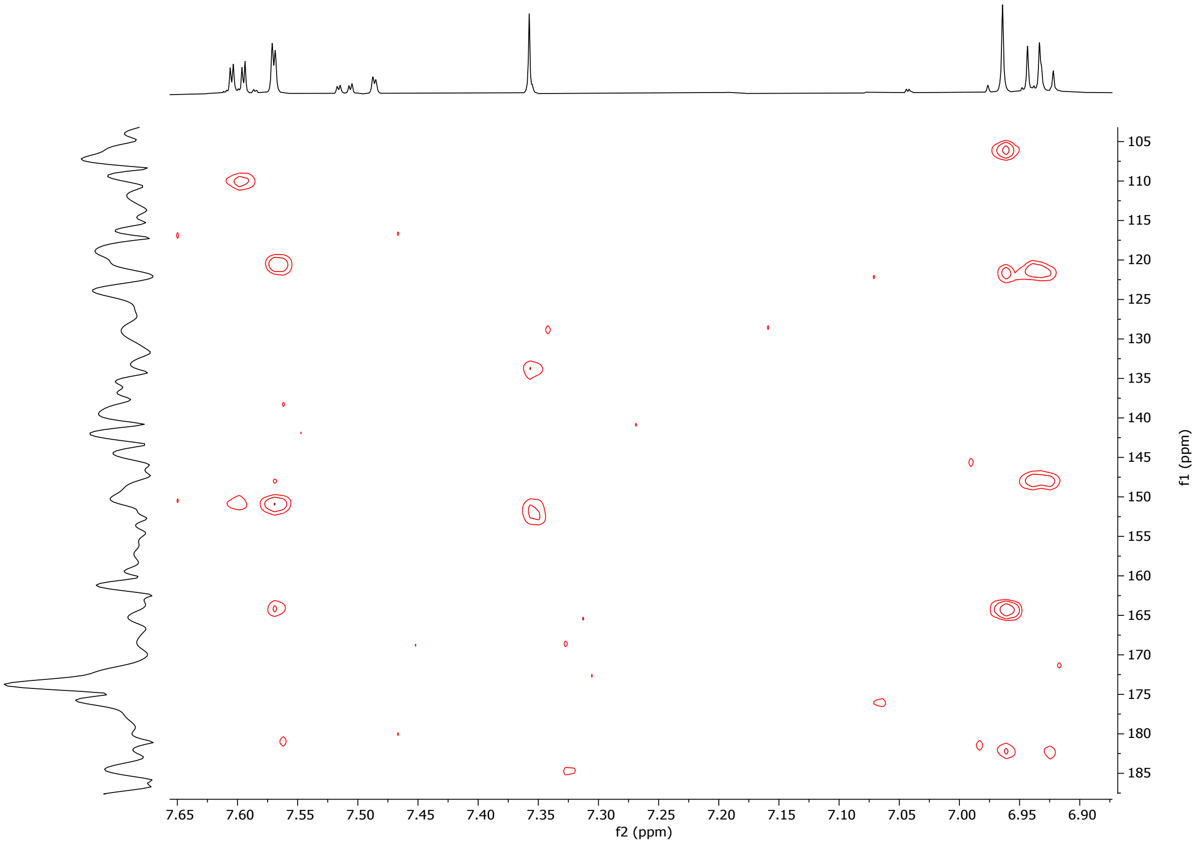

Figure S33: HMBC spectrum of **28** (DMSO-d_6).
